# SARS-CoV-2 Proteome Harbors Peptides Which Are Able to Trigger Autoimmunity Responses: Implications for Infection, Vaccination, and Population Coverage

**DOI:** 10.3389/fimmu.2021.705772

**Published:** 2021-08-10

**Authors:** Mohsen Karami Fath, Abolfazl Jahangiri, Mahmoud Ganji, Fatemeh Sefid, Zahra Payandeh, Zahra Sadat Hashemi, Navid Pourzardosht, Anahita Hessami, Maysam Mard-Soltani, Alireza Zakeri, Mohammad Reza Rahbar, Saeed Khalili

**Affiliations:** ^1^Department of Cellular and Molecular Biology, Faculty of Biological Sciences, Kharazmi University, Tehran, Iran; ^2^Applied Microbiology Research Center, Systems Biology and Poisonings Institute, Baqiyatallah University of Medical Sciences, Tehran, Iran; ^3^Department of Medical Biotechnology, Faculty of Medical Sciences, Tarbiat Modares University, Tehran, Iran; ^4^Department of Medical Genetics, Shahid Sadoughi University of Medical Sciences, Yazd, Iran; ^5^Immunology Research Center, Tabriz University of Medical Sciences, Tabriz, Iran; ^6^Advanced Therapy Medicinal Product (ATMP) Department, Breast Cancer Research Center, Motamed Cancer Institute, Academic Center for Education, Culture and Research (ACECR), Tehran, Iran; ^7^Biochemistry Department, Guilan University of Medical Sciences, Rasht, Iran; ^8^School of Pharmacy, Shiraz University of Medical Sciences, Shiraz, Iran; ^9^Department of Clinical Biochemistry, Faculty of Medical Sciences, Dezful University of Medical Sciences, Dezful, Iran; ^10^Department of Biology Sciences, Shahid Rajaee Teacher Training University, Tehran, Iran; ^11^Pharmaceutical Sciences Research Center, Shiraz University of Medical Sciences, Shiraz, Iran

**Keywords:** autoimmune disease, SARS-CoV-2, vaccination, peptide, HLA, population coverage

## Abstract

Autoimmune diseases (ADs) could occur due to infectious diseases and vaccination programs. Since millions of people are expected to be infected with SARS-CoV-2 and vaccinated against it, autoimmune consequences seem inevitable. Therefore, we have investigated the whole proteome of the SARS-CoV-2 for its ability to trigger ADs. In this regard, the entire proteome of the SARS-CoV-2 was chopped into more than 48000 peptides. The produced peptides were searched against the entire human proteome to find shared peptides with similar experimentally confirmed T-cell and B-cell epitopes. The obtained peptides were checked for their ability to bind to HLA molecules. The possible population coverage was calculated for the most potent peptides. The obtained results indicated that the SARS-CoV-2 and human proteomes share 23 peptides originated from ORF1ab polyprotein, nonstructural protein NS7a, Surface glycoprotein, and Envelope protein of SARS-CoV-2. Among these peptides, 21 peptides had experimentally confirmed equivalent epitopes. Amongst, only nine peptides were predicted to bind to HLAs with known global allele frequency data, and three peptides were able to bind to experimentally confirmed HLAs of equivalent epitopes. Given the HLAs which have already been reported to be associated with ADs, the ESGLKTIL, RYPANSIV, NVAITRAK, and RRARSVAS were determined to be the most harmful peptides of the SARS-CoV-2 proteome. It would be expected that the COVID-19 pandemic and the vaccination against this pathogen could significantly increase the ADs incidences, especially in populations harboring HLA-B*08:01, HLA-A*024:02, HLA-A*11:01 and HLA-B*27:05. The Southeast Asia, East Asia, and Oceania are at higher risk of AD development.

## Introduction

In 2019, the severe acute respiratory syndrome coronavirus 2 (SARS-CoV-2) caused a disease called coronavirus disease 2019 (COVID-19). It rapidly turned into a threat to global health and a progressive pandemic disease in many countries worldwide (1, 2). SARS-CoV-2 genome encodes for 14 open reading frames (Orfs). The Orf1a/Orf1ab encodes pp1a and pp1ab polyproteins which are further cleaved by virus-encoded proteases into 16 non-structural proteins (Nsps) (3, 4). The remaining Orfs encode structural proteins such as spike glycoprotein (S), the small envelope glycoprotein (E), the membrane glycoprotein (M), and a nucleocapsid protein (N) (3, 5). SARS-CoV-2 inters into the host cells *via* the interaction between the S glycoprotein of the virus and the angiotensin-converting enzyme 2 (ACE2) and the type II transmembrane serine protease (TMPRSS2) of the host cells. The S glycoprotein binds to the ACE2 receptor through its receptor-binding domain (RBD), which spans the 331 to 524 residues (4–7).

Autoimmune disease occurs when the body loses its immunological tolerance to its antigens (the failure of self-tolerance). ADs can appear anywhere in the body and three factors that induce and perpetuate autoimmune disease are hampered immune regulation, environmental factors, and genetic predisposition (8–11). Recent data have shown that environmental factors such as infectious agents (including viruses, bacteria, parasites, and fungi), dietary ingredients, and toxic Chemicals contribute more than 70% to loss of self-tolerance and, as result, autoimmunity (12–14). Autoimmune diseases could be induced by viruses (15). There are also some bacteria-induced autoimmune diseases (16). Moreover, immune responses to *Candida albicans* in peripheral blood lymphocytes and synovial fluids suggested that fungi may also lead to autoimmunity (17). Three major mechanisms of the infectious agents to trigger autoimmune diseases include bystander activation, epitope spreading, and molecular mimicry (12, 18). In the bystander mechanism, infected cells can activate uninfected-cells through intercellular communication (gap junctions), co-receptor expression [natural Killer group 2D (NKG2D), CD122, TLR (toll-like receptor)], and soluble signals (cytokines). Nonspecific activation of B and T cells is called bystander activation and is known by the activation of lymphocytes detached from the BCR/TCR specificity (18). Release of self-antigens during inflammatory or chronic autoimmune responses, can lead to autoimmune reactions against endogenous epitopes in a phenomenon known as epitope spreading (ES) (19). The ES can be induced by changes in protein structure, such as the conversion of arginine to citrulline. This change triggers an immune response to the original protein and its citrullinated form. Moreover, similar responses could be elicited against other citrullinated proteins; this mechanism is a characteristic of rheumatoid arthritis (RA). Pemphigus bullous, Systemic lupus erythematosus (SLE), pemphigoid, multiple sclerosis, and some other autoimmune diseases are all affected by intramolecular and intermolecular B cell epitope spreading. Somatic hyper-mutation, antigen presentation, and endocytic processing are the molecular mechanisms that support epitope spreading. They also enhance the immune response in ADs (20). Molecular mimicry is another mechanism of developing autoimmunity. This mechanism could lead to the activation of cross reactive T and, or B cells. It occurs when infectious agents contain foreign antigens similar in structure and sequence to the human self-antigens (21–23). This mechanism is implicated in the pathogenesis of many autoimmune diseases such as Graves’ disease, MS, spondyloarthropathies, and diabetes mellitus (24–26).

Recently, the association of various diseases with SARS-CoV-2 has been investigated, one of the most well-known of which are ADs. During the studies on ADs, the impact of SARS-CoV-2 in the progression of autoimmunity has been proven. Studies on the correlation of SARS-CoV-2 infection with diseases such as immune thrombocytopenic purpura (ITP), Miller Fisher syndrome (MFS), Kawasaki (KD), RA, Guillain-Barre syndrome (GBS), and SLE suggested that there is a connection between SARS-CoV-2 and autoimmune disorders. Infection with SARS-CoV-2 acts as a turning point for the progression of autoimmune disease. COVID-19 could reduce the threshold of immunological tolerance through molecular mimicry and epitope spreading (27, 28). The study for the feasibility of autoimmune responses against protein targets in SARS-CoV-2 infection demonstrates that different organs could be affected by anti-SARS-CoV-2 immune responses, and cytopathic effects could be directly induced. Moreover, the role of self-reactive antibodies in the infectious process of the viruses should not be overlooked (29). Another correlation study has been conducted on the lupus erythematosus. According to the results of this study, a significant increase in the levels of anti-SARS-CoV-2 antibody is visible in autoantibody-positive patients (50% of 21 ICU China patients & 92% of 11 ICU German patients) (30). A case report on a young woman with recurrent immune‐mediated lymphocytic fulminant myocarditis (FM) has also brought some evidence in to light. The outcome of this study points out that the autoimmune disease can activate or reactivate in patients with an immunogenic background *via* significant infections such as COVID-19. This means that predisposition to such genetic history can act as a powerful trigger for the immune system to respond beyond normal (31). It has also been revealed that the risk of COVID-19 is higher in the patients suffering from autoimmune diseases (32).

In the present study, we aimed to analyze the whole proteome of the SARS-CoV-2 for potential antigenic regions capable of triggering autoimmune responses. In silico studies have already been widely used to solve biological challenges (33–41). Various in silico tools have been harnessed to analyze the MHC2 binding epitopes of the SARS-CoV-2 proteome and assess their possible involvement in ADs. Moreover, the global HLA susceptibility map of SARS-CoV-2 for ADs was attained.

## Methods

### Study Flowchart

**Figure 1:**
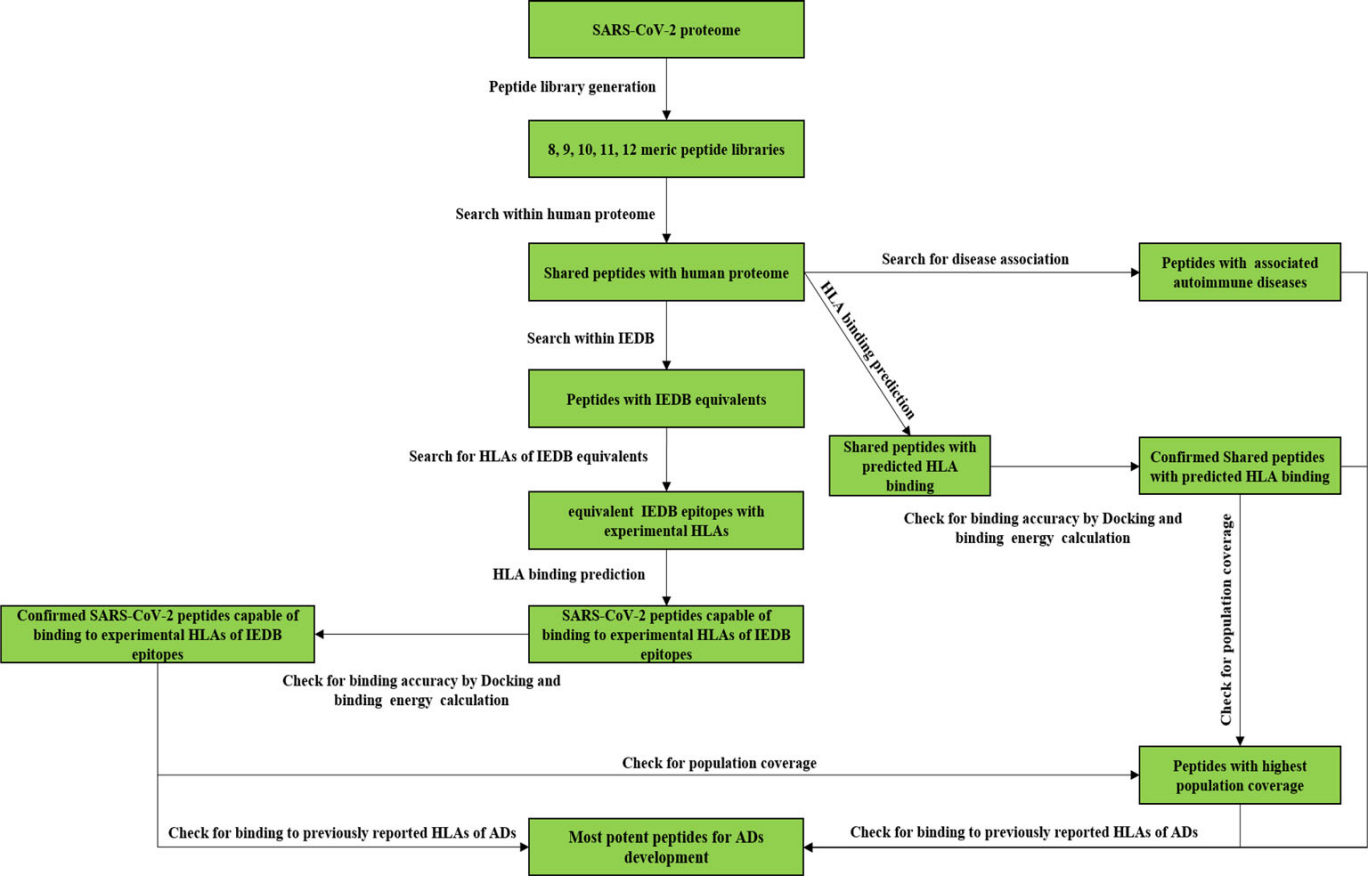
The study follow chart.

The study includes the analyses of more than 48000 peptides using various in silico tools to find out the most potent ADs inducing SARS-CoV-2 peptides. The designed procedure includes mutiple steps to gather reliable data about the ability of the SARS-CoV-2 proteome to trigger ADs following the COVID-19 or possible vaccination programs. The following diagram shows the study steps to get a better grasp of the analyses which would be conducted (**Figure 1**).

### Generation of Peptide Library for SARS-Cov-2 Proteome

The sequence for full polyprotein 1ab (ORF1ab), spike (S) protein, envelope (E) protein, nucleocapsid (N) protein, and membrane (M) protein, nonstructural protein NS3, nonstructural protein NS6, nonstructural protein NS7a, nonstructural protein NS7b, and nonstructural protein NS8 were obtained from the National Center of Biotechnology Information (NCBI) (https://www.ncbi.nlm.nih.gov/). The obtained sequences were confirmed with the sequences stored in the UniProtKB (https://www.uniprot.org/). The peptide generator server (https://www.peptide2.com/peptide_generator.php) was employed to dissect the viral proteome into 8mer, 9mer, 10mer, 11mer, and 12mer overlapping peptide libraries, while the overlapping amino acid count was set to be 7, 8, 9, 10, and 11, respectively.

### Peptide Similarity Search

The generated peptides for the SARS-CoV-2 proteome were checked against the human proteome to quickly retrieve all occurrences for a given query peptide from the UniProtKB protein sequences. The PIR Peptide Match tool was used to find the peptide matches. Employing the multiple peptide match interface of the PIR (https://research.bioinformatics.udel.edu/peptidematch/batchpeptidematch.jsp), the generated peptide libraries were checked against the human proteome [Homo sapiens [9606] (210556 seq.)] of the UniProtKB release 2020_05 plus isoforms (195,707,930 sequences). The search was set to include the isoforms to encompass all possibilities. In the case of the existing peptide match, the corresponding protein from the Human proteome was searched in UniProtKB. The information about the gene name, cellular functions, and protein-protein interaction network from the String server (https://string-db.org/) were extracted to analyze their possible correlation with autoimmune diseases.

### Prediction of Peptide-MHC Class I Binding Affinity

The Immune Epitope Database (IEDB) (http://www.iedb.org/) was searched to find equivalent epitopes similar to SARS-CoV-2 peptides. The search was restricted to epitopes which are experimentally tested for their ability to bind to adaptive immune receptors [T cell receptor (TCR), antibody or B cell receptor (BCR), or major histocompatibility complex (MHC)]. The search was also narrowed to include linear human epitopes with 90% identity to SARS-CoV-2 peptides. All of the SARS-CoV-2 peptides with a matching epitope in human proteome were assessed in IEDB search. The corresponding proteins for experimental IEDB epitopes were analyzed in the UniProt database. Moreover, the experimentally confirmed HLAs for equivalent IEDB epitopes and the involved disease were extracted from the IEDB epitope search results. The SARS-CoV-2 peptides with equivalent experimental IEDB epitopes were evaluated for their MHC class I-peptide binding affinity. These predictions were carried out against 145 different HLA alleles. These alleles were selected due to the availability of their global allele frequency data. The NetCTLpan 1.1 Server (http://www.cbs.dtu.dk/services/NetCTLpan/) was used to predict the CTL epitopes in the sequences of the selected peptides. The NetMHCpan 4.1 server (http://www.cbs.dtu.dk/services/NetMHCpan/) was used to predict the peptide-MHC class I binding using artificial neural networks (ANNs). Octamer peptides and the selected 145 HLA alleles were set to conduct the analyses. Moreover, the exact prediction was made for the selected SARS-CoV-2 peptides against the experimentally confirmed HLAs of equivalent IEDB epitopes.

### HLA Structures

The protein data bank (RCSB PDB) (https://www.rcsb.org/) and the PDBflex database (www.pdbflex.org) were used to find the 3D structures of the HLA molecules. The structure of the predicted HLAs for SARS-CoV-2 peptides and the structures of the experimentally determined HLAs for IEDB epitopes were obtained from the RCSB PDB. The structures resolved by the x-ray diffraction method, accompanied by a peptide, and had the highest resolution were selected for further evaluations. The Chimera 1.10.2 software was used to remove the redundant (peptide chains and the unwanted non-protein molecules) chains from the HLA structures. This would prepare them for the following docking analyses.

### Peptide Modeling

The structures of: (a) SARS-CoV-2 peptides with existing HLA predictions, and (b) the SARS-CoV-2 peptides predicted to bind to experimentally confirmed HLA molecules of the equivalent IEDB epitopes, were required to confirm their ability to bind to corresponding HLAs. Therefore, peptide modeling software was harnessed to determine the 3D structure of these peptides. The PEPSTRMOD server (http://osddlinux.osdd.net/raghava/pepstrmod/nat_ss.php) and PEP-FOLD 3.5 server (https://mobyle.rpbs.univ-paris-diderot.fr/cgi-bin/portal.py#forms::PEP-FOLD3) were employed to model the peptides. These servers are capable of modeling the peptides with natural amino acids. The quality of the modeled peptides was assessed using the QMEAN server (https://swissmodel.expasy.org/qmean/). The Protein Data Bank File Editor software was used to assign chain IDs for modeled epitopes.

### Molecular Docking

Molecular docking analysis was done for the modeled peptides with existing resolved HLA structures. The structures of the HLA molecules and the modeled peptides were used to perform the molecular docking study. The CABS-dock (http://biocomp.chem.uw.edu.pl/CABSdock), HPEPDOCK (http://huanglab.phys.hust.edu.cn/hpepdock/), and HADDOCK (https://wenmr.science.uu.nl/haddock2.4/) servers were used to perform the analyses. Both chains of the HLA molecule and the modeled peptides were set as the input molecules. Chimera software was used for visual inspection of the docked complexes to ensure their correct interaction orientation. FireDock (http://bioinfo3d.cs.tau.ac.il/FireDock/) refinement server utilizes a coarse refinement method to optimize the interaction in molecular docking studies. The software was used to refine the docked complexes. The results of the FireDock software were fed to rigid-body orientation and side-chain conformations optimization by the RosettaDock server (http://rosettadock.graylab.jhu.edu/).

### Binding Energy Calculation

Aside from the correct interaction orientation, the docked protein-peptide complexes should have strong binding energy to keep them together to properly present on the surface of immune cells. The PRODIGY (PROtein binDIng enerGY prediction) server (https://wenmr.science.uu.nl/prodigy/) was invoked to predict binding affinity in docked complexes. All of the docked complexes were subjected to this analysis. Moreover, the originally selected HLA complexes were checked for their binding affinity against their accompanying peptides as a positive control in comparison with the docked complexes.

### Data Validation and Disease Association Search

All identical peptides with human proteome were searched in IEDB to find similar experimentally validated epitopes. The corresponding HLA class I of experimental epitopes was evaluated *via* an integrated approach for the SARS-CoV-2 peptides. A literature review was conducted to invoke experimental studies as supporting evidence for SARS-CoV-2 and autoimmune disease association. The miPepBase database (http://proteininformatics.org/mkumar/mipepbase/index.html) is a database of experimentally verified peptides involved in molecular mimicry, which was used to find any Molecular Mimicry of matching peptides. Cross reactivity between the human “disease-related” epitopes and the matching peptides of the pathogen could trigger autoimmunity in a process, which is called molecular mimicry. On the other hand, the Gene and Autoimmune Disease association Database (GAAD) (http://gaad.medgenius.info/genes/) was employed to describe the possible association between genes and autoimmune disease. The gene IDs of the human proteins, which were obtained from the peptide match search, were used to run the analyses. The search would be carried out among the 4,186 genes which are found to associate with autoimmune diseases. It was important to know if the IEDB peptides were associated with any autoimmune diseases. Therefore, the miPepBase database was used to find epitopes with the ability to trigger Molecular Mimicry for autoimmune diseases. All of the IEDB epitopes found in the previous section were fed as input for these analyses. The possible association between the genes of the IEDB proteins and autoimmune disease were described by GAAD database. The gene IDs of the proteins (corresponding to the IEDB epitopes) were obtained from UniProt database. Moreover, a thorough literature review was also performed to find existing incidence of auto immune diseases following the COVID-19.

### Population Coverage

The population coverage analyses were done using the IEDB population coverage tool (http://tools.iedb.org/population/). This tool is used to calculate the fraction of individuals predicted to respond to a given epitope set based on HLA genotypic frequencies and on the basis of MHC binding and, or T cell restriction data. Two runs of population coverage analyses were executed for the obtained HLA and peptide sets. The first run was done for the SARS-CoV-2 peptides with existing HLA predictions. The second run was done for the SARS-CoV-2 peptides predicted to bind to experimentally confirmed HLA molecules of the equivalent IEDB epitopes. Moreover, to analyze the combined population coverage of all peptides and their predicted HLA molecules, another run was performed. The analyses were limited to the class one HLA molecules, and the selected areas and populations were set to include all of the possible areas and populations within the server.

## Results

### Peptide Library Generation

The genomic RNA sequences of full polyprotein 1ab (ORF1ab), spike (S) protein, envelope (E) protein, nucleocapsid (N) protein, membrane (M) protein, nonstructural protein NS3, nonstructural protein NS6, nonstructural protein NS7a, nonstructural protein NS7b, and nonstructural protein NS8 were found under the protein IDs of QHR63289.1, QHR63290.2, QHR63292.1, QHR63298.1, QHR63293.1, QHR63291.1, QHR63294.1, QHR63295.1, QHR63296.1, and QHR63297.1, respectively. All possible 8- to 12-mer peptides were generated from the SARS-CoV-2 proteome. 48530 peptides were generated and organized in 8mer, 9mer, 10mer, 11mer, and 12mer overlapping peptide libraries. Since the sliding window of peptide generation has one amino acid step size, each amino acid differed by one amino acid from its previous peptide.

### Matching Peptides in the Human Proteome

Searching for matching peptides among the human proteome sequences unveiled the existence of 23 SARS-CoV-2 peptides with exact matches within the human protein. All of the matching peptides were from the octamer library, and no other peptide libraries with different lengths had any matching peptides. Some of the peptides were found in more than one human protein. The list of SARS-CoV-2 octamer peptides and some information about the matching human proteins are presented in **Table 1**. The protein-protein interaction networks of the found proteins are presented in **Supplementary Figure 1**. Data regarding the tissue specificity of the found human proteins are represented in **Supplementary Table 1**. The corresponding proteins of Eight out of 23 SARS-CoV-2 peptides had been shown to be expressed within heart tissue.

**Table 1:** The results of peptide (octamers) similarity search between SARS-CoV-2 peptides and human proteome.

	Peptide	Source	UniProt ID	Protein ID	Protein Name	Length	String ID	Match Range
1	ESGLKTIL	ORF1ab polyprotein	P20073	ANXA7	Annexin A7	488	ENSP00000362010	FSGYVESGLKTILQCALN 404-411
2	EVEKGVLP	ORF1ab polyprotein	P52848	NDST1	Bifunctional heparan sulfate N-deacetylase/N-sulfotransferase 1	882	ENSP00000261797	VTRPSEVEKGVLPGEDWT 214-221
3	DEDEEEGD	ORF1ab polyprotein	Q96IK5	GMCL1	Germ cell-less protein-like 1	515	ENSP00000282570	PDSETDEDEEEGDEQQRL 69-76
4	PDEDEEEG	ORF1ab polyprotein	Q6P1N0	C2D1A	Coiled-coil and C2 domain-containing protein 1A	951	ENSP00000313601	LCMRDPDEDEEEGTDEDD 84-91
5	DIQLLKSA	ORF1ab polyprotein	O00423	EMAL1	Echinoderm microtubule-associated protein-like 1	815	ENSP00000334314	QMQEDDIQLLKSALADVV 51-58
6	EVLLAPLL	ORF1ab polyprotein	Q66PJ3-6	AR6P4	ADP-ribosylation factor-like protein 6-interacting protein 4	276	ENSP00000306788	TAPGAEVLLAPLLPPRPP 237-244
7	YNYEPLTQ	ORF1ab polyprotein	Q9UJA3	MCM8	DNA helicase MCM8	840	ENSP00000368164	IHARVYNYEPLTQLKNVR 199-206
8	RRSFYVYA	ORF1ab polyprotein	Q86W33	TPRA1	Transmembrane protein adipocyte-associated 1	373	ENSP00000347748	ISLPSRRSFYVYAGILAL 225-232
9	AKKNNLPF	ORF1ab polyprotein	Q92604	LGAT1	Acyl-CoA: lysophosphatidyl glycerol acyltransferase 1	370	ENSP00000355964	TSQAFAKKNNLPFLTNVT 199-206
10	DTSLSGFK	ORF1ab polyprotein	Q9Y666	S12A7	Solute carrier family 12 member 7	1083	ENSP00000264930	KYRSRDTSLSGFKDLFSM 995-1002
11	SLKELLQN	ORF1ab polyprotein	Q92674	CENPI	Centromere protein I	756	ENSP00000362018	CSVLQSLKELLQNWLLWL 496-503
12	PGSGVPVV	ORF1ab polyprotein	P19021	AMD	Peptidyl-glycine alpha-amidating monooxygenase precursor	973	ENSP00000306100	KLIKEPGSGVPVVLITTL 860-867
13	RYPANSIV	ORF1ab polyprotein	O95415	BRI3	Brain protein I3	125	ENSP00000313601	SRTVTRYPANSIVVVGGC 66-73
14	GPPGTGKS	ORF1ab polyprotein	O75351	VPS4B	Vacuolar protein sorting-associated protein 4B	444	ENSP00000297290	GILLFGPPGTGKSYLAKA 174-181
			Q7Z333	SETX	Probable helicase senataxin	2677	ENSP00000238497	ICLIHGPPGTGKSKTIVG 1963-1970
			Q9UN37	VPS4A	Vacuolar protein sorting-associated protein 4A	437	ENSP00000224140	GILLFGPPGTGKSYLAKA 167-174
15	NVAITRAK	ORF1ab polyprotein	P51530	DNA2	DNA replication ATP-dependent helicase/nuclease DNA2	1060	ENSP00000351185	DWRRLNVAITRAKHKLIL 1001-1008
16	QGPPGTGK	ORF1ab polyprotein	Q92900	RENT1	Regulator of nonsense transcripts 1	1129	ENSP00000470142	PLSLIQGPPGTGKTVTSA 502-509
			Q9BYK8	HELZ2	Helicase with zinc finger domain 2	2649	ENSP00000417401	PFTVIQGPPGTGKTIVGL 2173-2180
			Q9P2E3	ZNFX1	NFX1-type zinc finger-containing protein 1	1918	ENSP00000379412	ELAIIQGPPGTGKTYVGL 618-625
17	RFNVAITR	ORF1ab polyprotein	Q9BXT6	M10L1	RNA helicase Mov10l1	1211	ENSP00000262794	LSNSKRFNVAITRPKALL 1131-1138
18	VTLIGEAV	ORF1ab polyprotein	P52209	6PGD	6-phosphogluconate dehydrogenase, decarboxylating	483	ENSP00000270776	EYGVPVTLIGEAVFARCL 278-285
19	LALITLAT	nonstructural protein NS7a	P28222	5HT1B	5-hydroxytryptamine receptor 1B	390	ENSP00000358963	LLVMLLALITLATTLSNA 56-63
20	DEDDSEPV	Surface glycoprotein	Q9Y6X6	MYO16	Unconventional myosin-XVI	1858	ENSP00000401633	AAPPGDEDDSEPVYIEML 1404-1411
21	RRARSVAS	Surface glycoprotein	P37088	SCNNA	Amiloride-sensitive sodium channel subunit alpha	669	ENSP00000353292	PPHGARRARSVASSLRDN 201-208
22	VFLLVTLA	Envelope protein	Q6P4F1-2	FUT10	Alpha- (1,3)-fucosyltransferase 10	520	ENSP00000332757	CVTATVFLLVTLALDTVE 20-27
23	VNSVLLFL	Envelope protein	O60518	RNBP6	Ran-binding protein 6	1105	ENSP00000259569	ILDETVNSVLLFLQDPHP 409-416

### MHC-Binding Prediction

The IEBD search indicated that 21 (out of 24 SARS-CoV-2) peptides have experimentally confirmed equivalent epitopes with at least 90% of similarity. There was no exact match between the selected SARS-CoV-2 peptides and the IEBD epitopes. Moreover, there were no predicted posttranslational modifications for SARS-CoV-2 peptides. Since IEDB epitopes are tested for binding to an adaptive immune receptor, the SARS-CoV-2 peptides with over 90% sequence similarity could be expected to show similar immunological outcomes. The corresponding proteins, experimental HLAs, and involved diseases for equivalent IEDB epitopes are presented in **Supplementary Table 2**. Selected SARS-CoV-2 peptides were also checked against 145 HLAs with known global allele frequency data. The obtained results indicated that some of the selected SARS-CoV-2 peptides are capable of binding to these HLA molecules. It has been predicted that the peptides including the ESGLKTIL (binds to: HLA-B*08:01), EVLLAPLL (binds to: HLA-B*51:07), NVAITRAK (binds to: HLA-A*34:02), RYPANSIV (binds to: HLA-A*24:02, HLA-A*24:03, HLA-A*24:07, HLA-C*14:02, and HLA-C*14:03), RRSFYVYA (binds to: HLA-B*27:02, HLA-B*27:03, HLA-B*27:04, and HLA-B*27:05), and RFNVAITR (binds to: HLA-A*33:03 and HLA-A*74:01) are the only SARS-CoV-2 peptides predicted to bind to some of the examined HLA alleles. The capability to bind to HLA molecule indicates that these epitopes are potentially immunogenic. The results of the similar prediction for selected SARS-CoV-2 peptides against the experimentally confirmed HLAs of equivalent IEDB epitopes indicated that the peptides including the RRSFYVYA (binds to: HLA-B*27:05), NVAITRAK (binds to: HLA-A*11:01), and RFNVAITR (binds to: HLA-A*31:01) could bind to examined HLA molecules.

### Preparation of HLA 3D Structures

It is vitally important to gather structural information about the interactions between an epitope and its HLA molecule. The 3D structures of some HLA molecules are already resolved and stored in protein structure databases. The search within the RCSB PDB indicated that four cases of the predicted HLAs for SARS-CoV-2 peptides and 61 cases of experimentally determined HLAs for IEDB epitopes have 3D structures. Some of the HLA molecules had more than one resolved structure. The structure with the highest resolution was selected for further analyses. The structures of the accompanying epitopes were removed from the chosen HLA structures. **Supplementary Table 3** listed the RCSB PDB IDs for the HLA molecules, found with a resolved 3D structure. The accompanying peptides of the HLA complexes were removed from the peptide-binding groove for the following docking analyses.

### Peptide Modeling

The structures of 4 peptides were modeled by the PEPSTRMOD and FOLD 3.5 servers. All of the peptides were correctly folded. The model quality assessment showed that the modeled peptides are of high structural quality. The modeled peptides were assigned with C or D chain IDs to be distinguished from the HLA chain IDs upon the docking analyses.

### Molecular Docking

The employed CABS-dock, HPEPDOCK, and HADDOCK software could dock the HLA structures with the modeled epitopes. All of the resulting complexes were visually inspected for their correct interactions. The obtained results indicated that the peptides were docked within the peptide-binding groove of the HLA molecules. The FireDock and RosettaDock servers optimized the docking orientation and side-chain conformations to have more reliable complexes of HLA and peptide molecules (**Supplementary Figure 2**). The obtained results demonstrated that the SARS-CoV-2 peptides could interact with the experimentally confirmed HLA molecules.

### Binding Energy Calculation

The results of binding energy calculation indicated that all of the modeled peptides can bind to the HLA molecules with an affinity equivalent to the binding affinity of resolves HLA-peptide complexes (**Table 2**). The predicted HLA (HLA-B*08:01 under the PDB ID of 3X13) showed the highest binding affinity (-13.7 kcal mol-1) against the corresponding SARS-CoV-2 peptide (ESGLKTIL). This binding affinity was even higher than the binding energy of the peptide and HLA molecule within the original 3X13 complex and the binding energy between the similar IEDB peptides and their HLA molecules. This high binding energy could be construed as the high potency of these peptides to invoke a robust immune response.

**Table 2:** The binding energy between the modeled peptides and the HLA molecules.

Peptide	HLA	PDB Code	ΔG (kcal mol^-1^)	K_d_ (M) at 25.0°C
ESGLKTIL	HLA-B*08:01	3X13	-13.7	8.9E-11
CONTROL	HLA-B*08:01	3X13	-10.9	9.9E-09
RYPANSIV	HLA-A*24:02	3WLB	-12.8	4.0E-10
CONTROL	HLA-A*24:02	3WLB	-11.4	4.5E-09
RRSFYVYA	HLA-B*27:05	5IB2	-12.7	5.0E-10
CONTROL	HLA-B*27:05	5IB2	-10.9	1.0E-08
NVAITRAK	HLA-A*11:01	4N8V	-12.4	7.8E-10
CONTROL	HLA-A*11:01	4N8V	-11.0	8.4E-09

### Disease Association Search

The SARS-CoV-2 octamer peptides with matching peptides in human proteome were analyzed for their association with autoimmune diseases. The results of the search within miPepBase database showed that some of the peptides could trigger the molecular mimicry mechanism and lead to autoimmune diseases. The results of the miPepBase database are presented in **Table 3**. The possible association between the found human protein matches and the autoimmune disease was analyzed using the GAAD server. The gene names for all of the matching proteins were extracted and used for the analyses. The obtained results revealed that only the SARS-CoV-2 peptide, which matches with the GMCL1 gene name, could be associated with Multiple sclerosis and Rheumatoid arthritis. The rest of the peptides had no associated diseases. The literature review results showed that 21 autoimmune conditions could be triggered following the COVID-19 (**Table 4**). The literature review results encompasses the results of the miPepBase and GAAD databases.

**Table 3:** The results of the miPepBase. All of the peptides with matching human proteins are analyzed.

	Peptide	UniProt ID	Protein name	Mimicry Peptide Sequence	Disease caused
**1**	EVEKGVLP	P10809	HSP60	HRKPLVIIAEDVDGE	Rheumatoid arthritis
**2**	DEDEEEGD	P10809	HSP60	HRKPLVIIAEDVDGE	Rheumatoid arthritis
**3**	EVLLAPLL	Q14008	Colonic TOG	GPSLR	Corhns disease
**4**	YNYEPLTQ	P14410	intestinal sucrose iso-maltase	KLNRIPS	Corhns disease
**5**	AKKNNLPF	P12883	cardiac myosin	LEDLKRQLEEEVKAKNA	Rheumatic carditis
		P14410/	intestinal sucrose isomaltase	KLNRIPS	Corhns disease
**6**	PGSGVPVV	P30493	HLA antigens	AQAQTDRESL	Ankylosing spondylitis
		P30488	HLA antigens	TNTQTYRESL	Ankylosing spondylitis
		P03989	HLA antigens	AKAQTDREDL	Ankylosing spondylitis
		C5MK52	HLA antigens	AKAQTDREDL	Ankylosing spondylitis
		A0A0k0kRG3	HLA antigens	AKAQTDRENL	Ankylosing spondylitis
		P01892	HLA antigens	AHSQTHRVDL	Ankylosing spondylitis
		P01891	HLA antigens	AQSQTDRVDL	Ankylosing spondylitis
**7**	GPPGTGKS	P02686	MBP	ENPVVHFFKNIVTPR	Multiple sclerosis
		F6RT34	MBP	VVHFFKNIVTP	Multiple sclerosis
		C9J6H1	MBP	VVHFFKNIVTP	Multiple sclerosis
**8**	RFNVAITR	P13533	cardiac myosin	LEDLKRQLEEEVKAKNA	Rheumatic carditis
		P12883	cardiac myosin	KLQTENGE	Rheumatic carditis

**Table 4:** The literature review results for autoimmune conditions triggered following the COVID-19.

	Autoimmune Disease	Reference
1	Antiphospholipid syndrome	(42–44)
2	Guillain-Barr´e syndrome	(45–47)
3	Miller Fisher syndrome	(48, 49)
4	Polyneuritis cranialis	(49)
5	Thyroid function	(50, 51)
6	Graves’ disease	(52)
7	Vasculitis	(53)
8	Kawasaki disease	(54)
9	Type 1 Diabetes	(55–57)
10	Autoimmune hemolytic anemia	(58–60)
11	Immune thrombocytopenic purpura	(61, 62)
12	Systemic lupus erythematosus	(63, 64)
13	Post orthostatic tachycardia syndrome	(65)
14	Viral arthritis	(66, 67)
15	Myasthenia gravis	(68)
16	Autoimmune encephalitis	(69)
17	Rheumatoid arthritis	(70, 71)
18	Autoimmune limbic encephalitis	(72)
19	Multiple sclerosis	(73, 74)
20	Inflammatory Bowel Disease (Crohn’s disease and ulcerative colitis)	(75)
21	Ankylosing spondylitis	(76)

### Population Coverage Analyses

**Figure 2:**
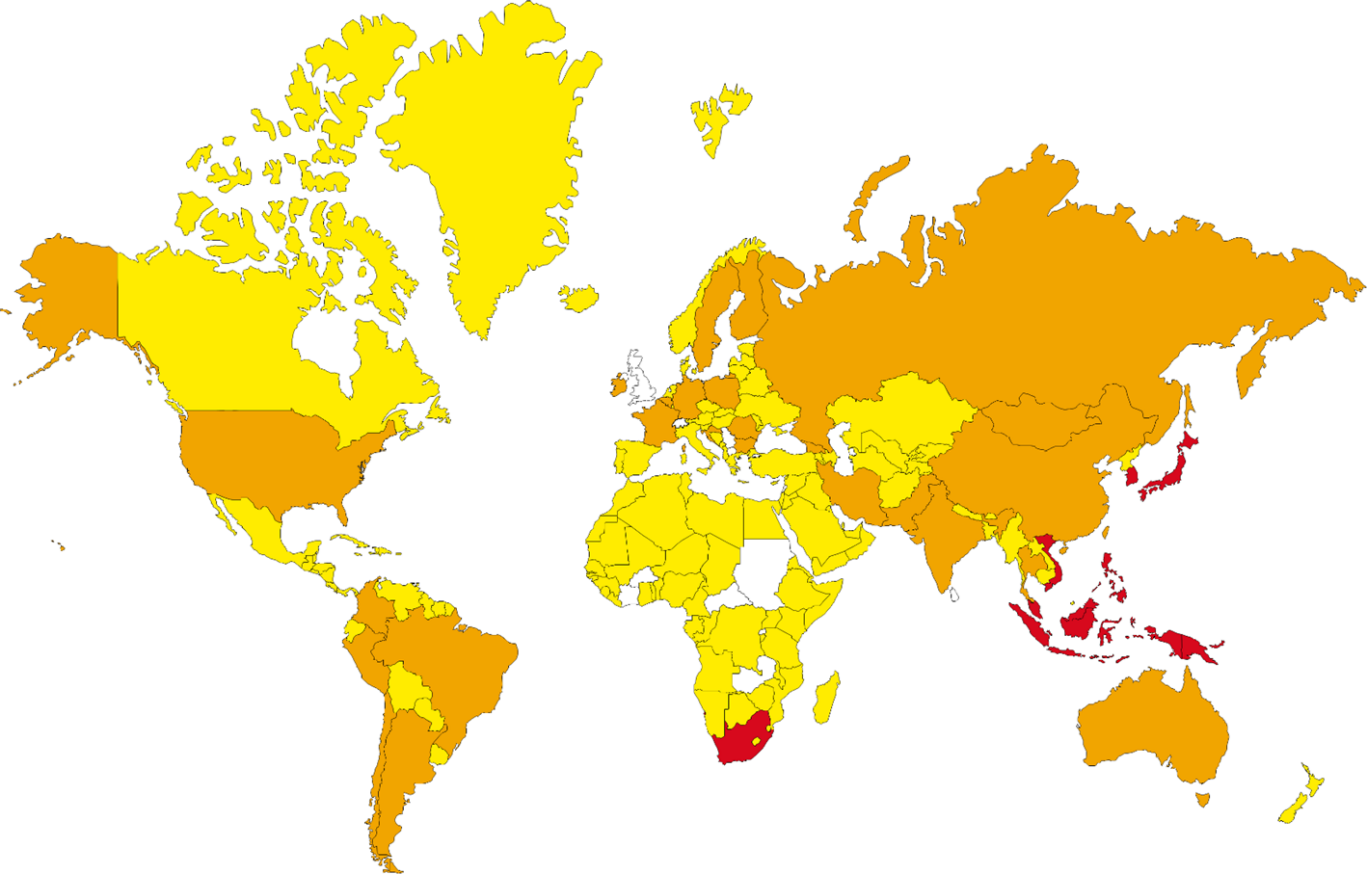
The population coverage prediction for most potent SARS-CoV-2 peptides (red: 100%-75%, orange: 75%-50%, yellow: 50%-25% and regions without data, white: 25%-0% of coverage).

Performing the population coverage analyses, the possible world coverage of each SARS-CoV-2 peptide for autoimmune responses was evaluated (**Table 5**). The attained results indicated that regarding the peptides with the highest population coverages, the RYPANSIV (world coverage 25.74%), NVAITRAK (world coverage 15.53%), ESGLKTIL (world coverage 10.55%), RRSFYVYA (world coverage 7.33%), and RFNVAITR (world coverage 6.91%) are the top five peptides. On the other hand, calculation of combined population coverage for all peptides with predicted HLA binding indicates over 57% world coverage (**Supplementary Table 4**). Among different world regions the Southeast Asia (coverage: 84.12%), East Asia (coverage: 83.78%), and Oceania (coverage: 80.72%) have the highest population coverages. Europe, North America, and South America have over 50 % coverage, while Africa is below 50% coverage. On the contrary, the regions like Lebanon, United Kingdom, and Rwanda have been calculated to have the lowest population coverage for the given peptide and HLA sets (**Figure 2**).

**Table 5:** The population coverage calculation for peptides with predicted HLA binding.

Peptide	HLA allele	World population coverage
**Selected SARS-CoV-2 peptides against 145 HLAs with known global allele frequency**		
ESGLKTIL	HLA-B*08:01	10.55%
EVLLAPLL	HLA-B*51:07	0.12%
NVAITRAK	HLA-A*34:02	0.5%
RYPANSIV	HLA-A*24:02	21.38%
RYPANSIV	HLA-A*24:03	0.49%
RYPANSIV	HLA-A*24:07	1.04%
RYPANSIV	HLA-C*14:02	3.04%
RYPANSIV	HLA-C*14:03	0.87%
RRSFYVYA	HLA-B*27:02	1.58%
RRSFYVYA	HLA-B*27:03	0.24%
RRSFYVYA	HLA-B*27:04	0.79%
RRSFYVYA	HLA-B*27:05	4.78%
RFNVAITR	HLA-A*33:03	4.78%
RFNVAITR	HLA-A*74:01	2.18%
RYPANSIV	HLA-A*24:02, HLA-A*24:03, HLA-A*24:07, HLA-C*14:02, and HLA-C*14:03	25.74%
RRSFYVYA	HLA-B*27:02, HLA-B*27:03, HLA-B*27:04, and HLA-B*27:05	7.33%
RFNVAITR	HLA-A*33:03 and HLA-A*74:01	6.91%
**Selected SARS-CoV-2 peptides against the experimentally confirmed HLAs of equivalent IEDB epitopes**		
RRSFYVYA	HLA-B*27:05	5.36%
NVAITRAK	HLA-A*11:01	15.53%
RFNVAITR	HLA-A*31:01	5.36%
**All peptides against all predicted HLAs**		
All peptides	all predicted HLAs	57.41%

## Discussion

Infections could trigger host immune responses against their immunogenic epitopes. Among these responses, some immunological cross-reactions against the epitopes of host proteins could occur (23). Epidemiological studies revealed that infectious diseases could trigger autoimmunity *via* cross-reactivity. Immunological mechanisms such as molecular mimicry and significant homology between microbial and human antigens could break off the self-tolerance and consequently induce the post-infection ADs. However, these cross-reactions could act as a double-edged sword in autoimmunity (23). Some reports have already suggested that a correlation exists between the COVID-19 and autoimmune diseases (77). Meanwhile, vaccines’ safety regarding their potential to trigger autoimmune responses remains a challenging issue (78). Molecular mimicry between the host antigens and the infectious agent or between the host antigens and the vaccine is a leading mechanism to induce autoimmunity (23, 78).

To find peptide matches within the human proteome, the proteome of SARS-CoV-2 was chopped to peptides with different lengths (8-12mer). Among the various lengths of HLA binders, only the shortest lengths of HLA class I binders (8-meric peptides) matched with peptides in the human proteome. This length of the peptide could also be considered as a B-cell epitope. Therefore, peptides with this length and their related HLAs (HLA class I) were considered for further analyses. Following the 2009 H1N1 pandemic in which tens of millions of vaccine doses against H1N1 have been delivered, reports have revealed an increased incidence of narcolepsy and Guillain-Barre syndrome (GBS) (23). This increase was attributed to epitope mimicry, and the genetic susceptibility of populations that received the vaccines. Kanduc et al. have revealed that two octapeptides of human proteome are shared with HPV16 proteome (79). HPV vaccination remains to be a challenging issue regarding the induction of ADs (23). Since the number of shared octapeptides between the human and SARS-CoV-2 proteome is more than 11-fold compared to the HPV16 and human proteome, the risk of triggering ADs could be significantly higher *via* SARS-CoV-2 infection and, or vaccination. In this regard, the study by Venkatakrishnan et al. has arrived at some epitopes with possible autoimmune consequences (80). Hence, subunit vaccines lacking these peptides would be safer concerning ADs development.

The SARS-CoV-2 proteome contains four proteins with matching peptides identical to peptides from the human proteome. Amongst, ORF1ab polyprotein encompasses the highest number of these peptides (18 peptides). Thus, this protein would outweigh other SARS-CoV-2 antigens regarding the risk of autoimmune responses. Aside from significant homology between pathogen and human peptides, genetic (e.g., some HLA alleles) and environmental factors (e.g., adjuvant administration) are involved in autoimmunity etiology. Finding positive experimental assays for SARS-CoV-2 epitopes assures the elicitation of the immune system against these peptides. Moreover, positive experimental assays for self-epitopes and HLA-binders, sharing identity with 8-meric peptides (identical to human proteome) increase the risk of cross-reactivity. However, peptides with “perfect fit” and identical homology are not necessarily more pathogenic than peptides with “almost identical” homology (23). Various counterexamples have already been reported for peptides of “almost identical” homology with higher pathogenicity (81, 82). Apparently, identical/similar peptides of infectious agents would be more harmful if they are assigned as binders of HLAs, which have already been determined to be involved in autoimmune diseases. It has been demonstrated that several HLAs are highly associated with ADs (e.g., A*24, A*68, B*08, B*15, B*27, B*42, B*51 and DRB1) (81, 83–86). Various mechanisms such as alternate docking, altered peptide-binding register, and hotspot molecular mimicry had been described for autoreactive T cell activation *via* HLAs-mediated peptide presentation (81). Therefore, the most deleterious epitopes share sequence identity with experimentally validated self-epitopes and bind to HLAs involved in autoimmune diseases. All of the SARS-CoV-2, predicted to be HLA binders, could potentially bind to HLAs involved in autoimmune diseases, among which five peptides were derived from the ORF1ab polyprotein.

The ESGLKTIL peptide was found within a 9-meric epitope of Annexin 7. Based on experimental assays, this epitope could bind to HLA-B*40:01 and HLA-B*40:02. Moreover, ESGLKTIL was also found within a 9-meric epitope of SARS-CoV-2, which is known as an HLA-B*40:01 binder. Although HLA-B*40:01 and HLA-B*40:02 are protective for autoimmune hepatitis (87), these alleles are associated with ankylosing spondylitis (AS) (88, 89). Ankylosing spondylitis is associated with SARS-CoV-2 (76).Besides, positive experimental assays revealed that ESGLKTIL shares sequence identity (more than 90%) with 11-meric and 16-meric peptides from phosphoglycolate phosphatase. These two peptides are reported to respectively bind to HLA-B*57:01 and HLA-F*01:03. It has been demonstrated that HLA-B*57:01 could be susceptible to drug-induced autoimmunity. Abacavir is a carbocyclic 2′-deoxyguanosine nucleoside analog which is suggested to be involved in loading novel self-peptides onto HLA-B*57:01 (90). A non-classical HLA, the HLA-F*01:03, could be associated with ankylosing spondylitis (91). The other autoimmune-related HLAs predicted to bind the SARS-CoV-2 are the HLA-B*08:01, HLA-A*24, and HLA-B*51. The HLA-B*08:01 is involved in myasthenia gravis, while the HLA-A*24 and HLA-B*51 are associated with Behçet disease** **(BD). Therefore, populations harboring these alleles are highly at risk of ADs following SARS-CoV-2 infection and/or vaccination.

The RYPANSIV peptide could bind to the HLA-A*024:02. This allele is associated with type 1 diabetes (92), myasthenia gravis (93), Sjogren’s syndrome (94), and systemic lupus erythematosus (95). Evidences are available about association of SARS-CoV-2 and systemic lupus erythematosus (63, 64). Moreover, this peptide shares identity with human peptides known as binders of autoimmune-associated HLAs. NVAITRAK is a binder of HLA-A*11:01, which is associated with autoimmune and inflammatory diseases such as type 1 diabetes (92), diffuse panbronchiolitis (96), and otosclerosis (97). Various studies revealed that SARS-CoV-2 is associated with type 1 diabetes (55–57). The RRARSVAS peptide is a binder for HLA-B*27:05. This allele is a well-known HLA associated with ankylosing spondylitis (98).

The RRARSVAS peptide is found in a neutralizing B-cell epitope of SARS-CoV-2 surface glycoprotein (99). Moreover, RRARSVAS is located near to motif responsible for super-antigenicity property of the spike protein. This epitope is located close to the S1/S2 cleavage site of the spike protein and resembles the super-antigen motif from *Staphylococcal enterotoxin B* (SEB). This sequence could be the possible explanation for cytokine storm in severe COVID-19 patients and multisystem-inflammatory syndrome (MIS-C) observed in children (100, 101). This sequence is also found in T-cell epitopes of SARS-CoV-2 spike protein. These T-cell epitopes are known to bind to various HLAs, including those associated with ADs (HLA-B*08:01 and HLA-B*07:02) (102–104). Moreover, the RARSVA sequence is found in an 11-meric epitope derived from a human antigen. This 11-meric epitope is known to bind to HLA-A*66:01 allele. The HLA-B*08:01 is associated with myasthenia gravis (105), and the HLA-B*07:02 is associated with neurological ADs (85). Reports are available about myasthenia gravis associated with SARS-CoV-2 infection (68, 106–108). Surface glycoprotein is the leading antigen of interest for vaccine development against SARS-CoV-2 (109). DEDDSEPV peptide is conserved in B-cell epitopes of SARS-CoV-2 and SARS-CoV-1 surface glycoproteins (110–113). Moreover, this peptide has recently been found in a 10-meric T-cell epitope of SARS-CoV-2 spike protein. This epitope is reported to bind to HLA-B*40:01 allele (102). The DDSEPV sequence has also been found in an epitope derived from a human antigen. This epitope is known to bind to various HLAs, including those associated with ADs (e.g., HLA-B*27:05 and HLA-C*08:01) (114, 115). Hence, vaccine candidates harboring either of these two peptides (DEDDSEPV and RRARSVAS) could be harmful considering the ADs development. The RRARSVAS peptide could be assigned as the most harmful peptide, since it is found in a neutralizing B-cell epitope of SARS-CoV-2. Therefore, this epitope could be of interest in vaccine design and formulation.

The EVEKGVLP peptide shares > 66% identity with GLVEVEKGV, which is a 9-meric epitope of SARS-CoV-2. This epitope is known to bind to the HLA-A*02:01 allele. This HLA allele is associated with vitiligo autoimmune disease (116). The DIQLLK sequence of the DIQLLKSA peptide (from SARS-CoV-2) was found in one SARS-CoV-1 (GEDIQLLKA) and two human (KRDIQLLK and DIQLLKRTV) HLA binders. The GEDIQLLKA peptide could bind to HLA-B*44:03, which is associated with vitiligo (117). Another study has also shown its association with autoimmune encephalitis (AE) as a neurological autoimmune disease (118). Interestingly, HLA-B*44 has a permissive role in SARS-CoV-2 infection (119). Association of autoimmune encephalitis with COVID-19 was also reported (69). The HLA-B*18:01 allele is associated with an inflammatory thyroid disease known as subacute thyroiditis (SAT) (120). Recently, it has been demonstrated that SARS-CoV-2 could cause thyroid dysfunction (50, 51). As mentioned above, HLA-B*40:02 is associated with ankylosing spondylitis. The DIQLLKRTV peptide is an HLA-A*33:01 binder which is associated with vitiligo (117) and drug-induced liver injury (DILI) (121). Although no positive assays have confirmed the EVLLAPLL peptide as an epitope or HLA binder from SARS-CoV-2, LLAPLL sequence was found in human epitopes, which acts as HLA binders associated with ADs.

Data validation study showed that our predicted autoimmune concerns already have conforming clinical records. These data instances are mostly accumulated since 2020. More than 21 autoimmune conditions are reported to be followed by COVID-19. Although these are preliminary studies of possible role of COVID-19 in triggering or exacerbating the autoimmune conditions, this number shows the perturbing nature of possible consequences. There is even a compatible tissue specificity profile between the predicted epitopes and the resulting autoimmune condition. For instance, myocardial involvement is characteristic for children with MIS-C associated with previous SARS-CoV-2 infection (101). The Annexin 7 is reported to be expressed within the heart tissue. The presence of the ESGLKTIL peptide within this antigen could be related to this post COVID-19 observation. The Amiloride-sensitive sodium channel subunit alpha is also reported to be expressed within the heart tissue. The presence of the RRARSVAS peptide within this antigen could also be related to myocardial involvement in children with MIS-C. Future studies about the exact mechanism of autoimmunity following COVID-19 would shed light on the roles played by mimicking peptides. We believe that getting a better grasp of autoimmune consequences of these mimicking peptides could be drastically important when the threat is global. This is more prominent when the whole population of the world is the target for vaccination programs and the disease would most likely become a seasonal infection for the next decades.

In conclusion, it would be expected that pandemic COVID-19 and the vaccination against this pathogen could significantly increase ADs, particularly those associated with HLA-B*08:01, HLA-A*024:02, HLA-A*11:01, and HLA-B*27:05. Populations harboring these alleles are highly at risk for the associated ADs. ESGLKTIL, RYPANSIV, NVAITRAK, and RRARSVAS are the most harmful peptides of the SARS-CoV-2 proteome. These peptides are binders for HLA-B*08:01, HLA-A*024:02, HLA-A*11:01, and HLA-B*27:05, respectively which could cover a high percentage of different populations throughout the world. Given these peptides and corresponding HLAs, the populations of Southeast Asia, East Asia, and Oceania are predicted to be at higher risk of AD development following the SARS-CoV-2 infection or vaccination.

## Data Availability Statement

The original contributions presented in the study are included in the article/**Supplementary Material**. Further inquiries can be directed to the corresponding author.

## Author Contributions

All authors designed the analysis, carried out the experiment, and collected the data. All authors contributed to the article and approved the submitted version.

## Funding

This work was supported by Shahid Rajaee Teacher Training University under contract number 23273.

## Conflict of Interest

The authors declare that the research was conducted in the absence of any commercial or financial relationships that could be construed as a potential conflict of interest.

## Publisher’s Note

All claims expressed in this article are solely those of the authors and do not necessarily represent those of their affiliated organizations, or those of the publisher, the editors and the reviewers. Any product that may be evaluated in this article, or claim that may be made by its manufacturer, is not guaranteed or endorsed by the publisher.
