# Supplementary material for: SARS-CoV-2 Proteome Harbors Peptides Which Are Able to Trigger Autoimmunity Responses: Implications for Infection, Vaccination, and Population Coverage
Source: Front Immunol. 2021 Aug 10;12:705772. doi: 10.3389/fimmu.2021.705772 (PMC8383889; doi:10.3389/fimmu.2021.705772)
Supplement: Supplementary file 1 [file Image_1.pdf]

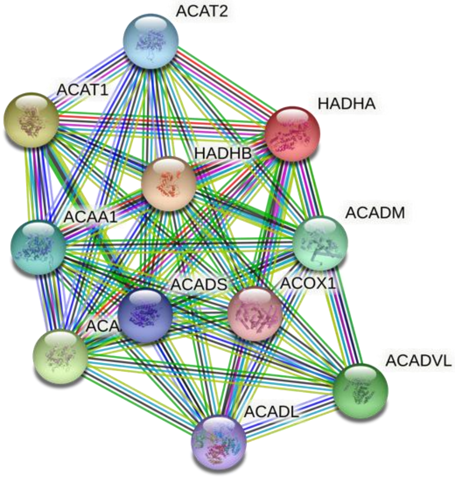

Trifunctional enzyme subunit alpha, mitochondrial precursor

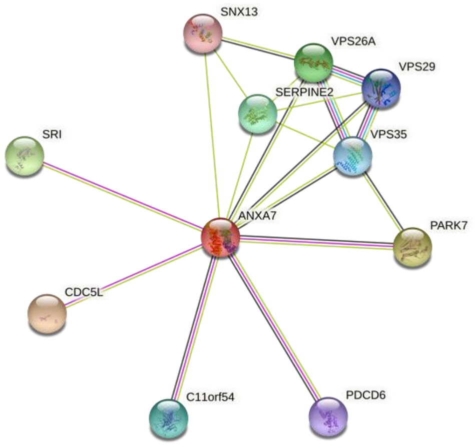

Annexin A7

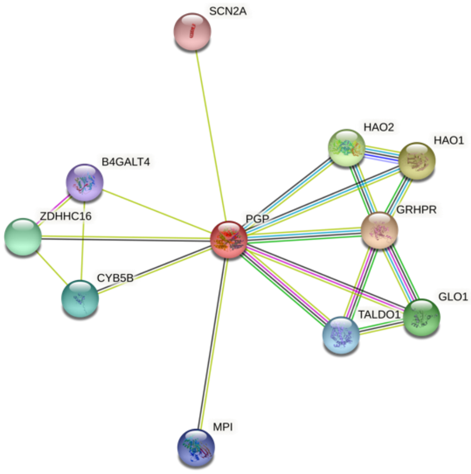

phosphoglycolate phosphatase

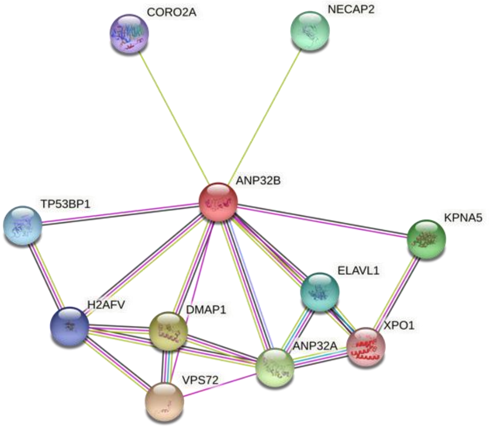

Acidic leucine-rich nuclear phosphoprotein 32 family member B

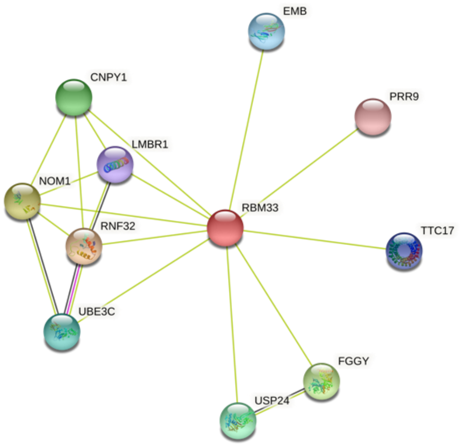

RNA-binding protein 33

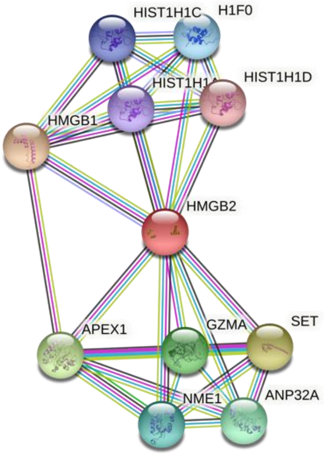

High mobility group protein B2

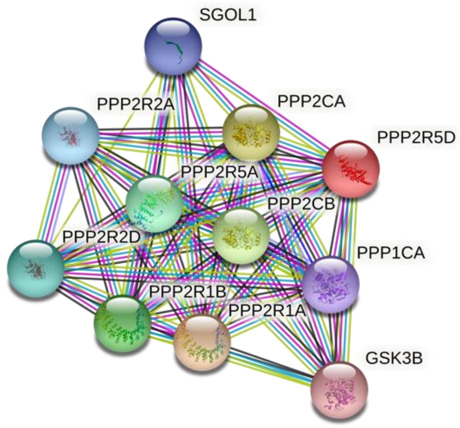

Serine/threonine-protein phosphatase 2A 56 kDa regulatory subunit delta isoform

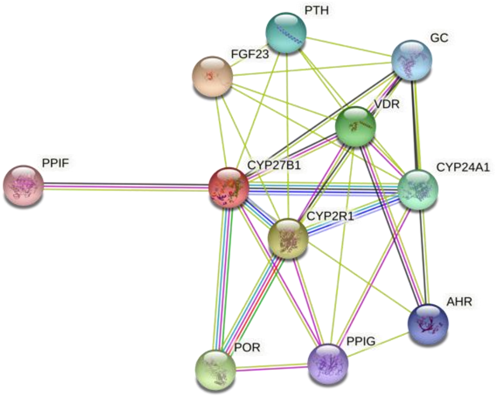

25-hydroxyvitamin D-1 alpha hydroxylase, mitochondrial

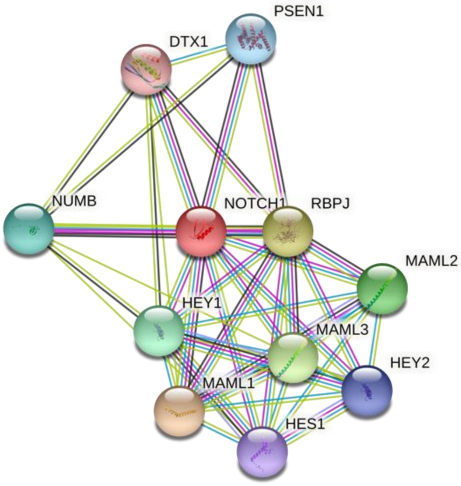

Neurogenic locus notch homolog protein 1

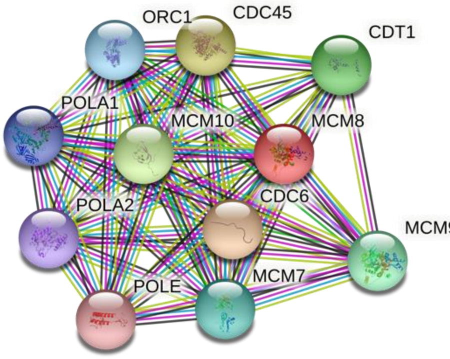

DNA helicase MCM8

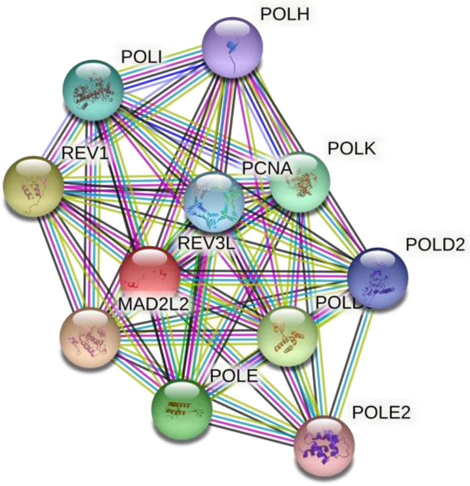

DNA polymerase zeta catalytic subunit

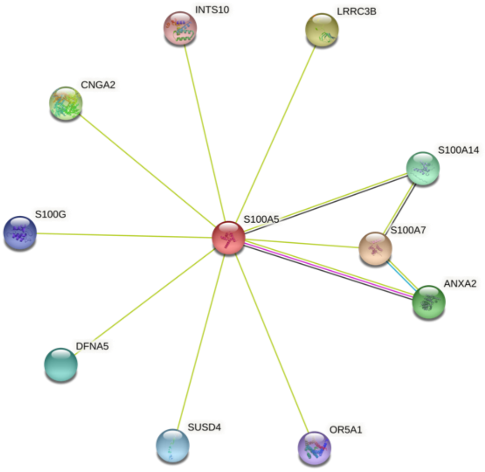

S100 calcium binding protein A1 isoform 2

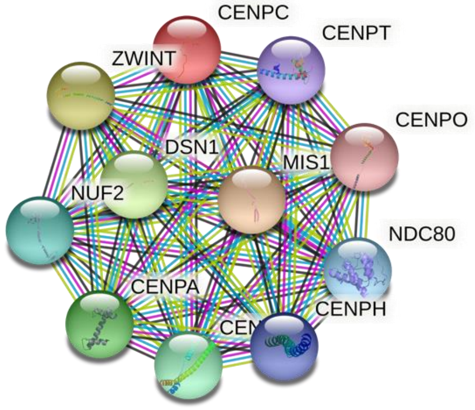

Centromere protein C

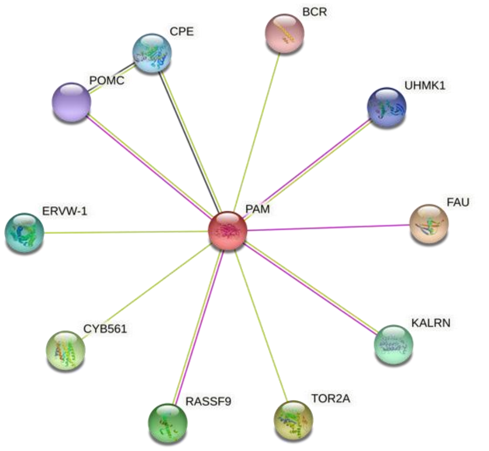

Peptidyl-glycine alpha-amidating monooxygenase precursor

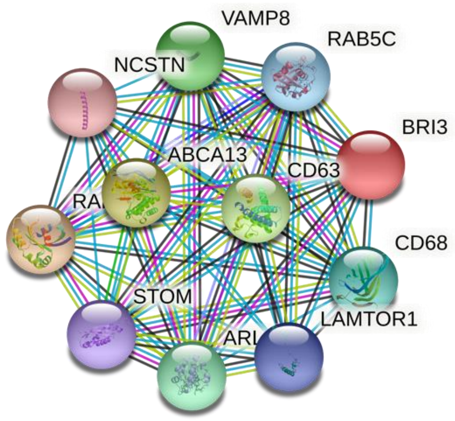

Brain protein I3

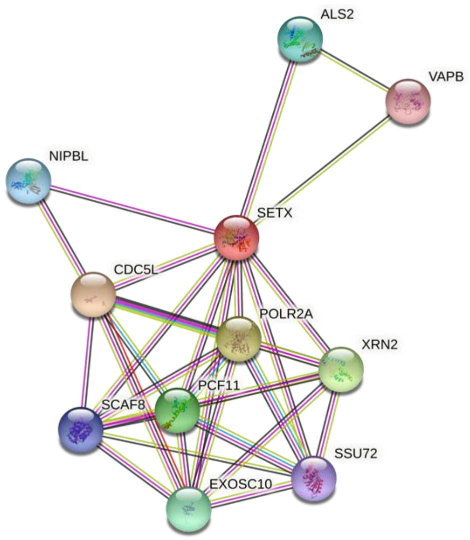

Probable helicase senataxin

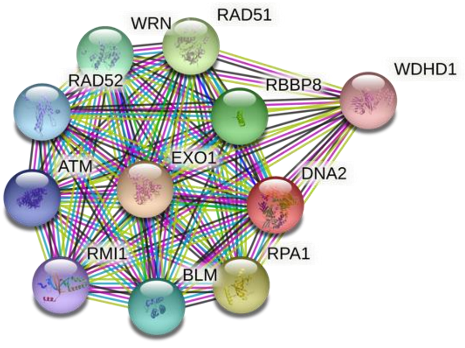

DNA replication ATP-dependent helicase/nuclease DNA2

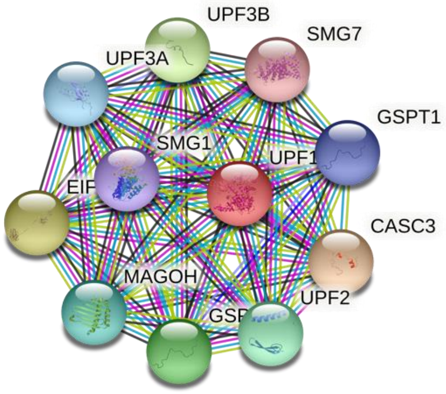

Regulator of nonsense transcripts 1

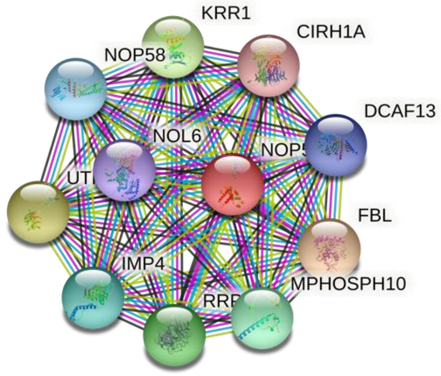

Nucleolar protein 56

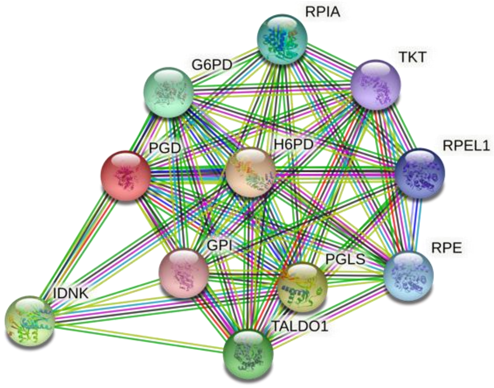

6-phosphogluconate dehydrogenase, decarboxylating isoform 2

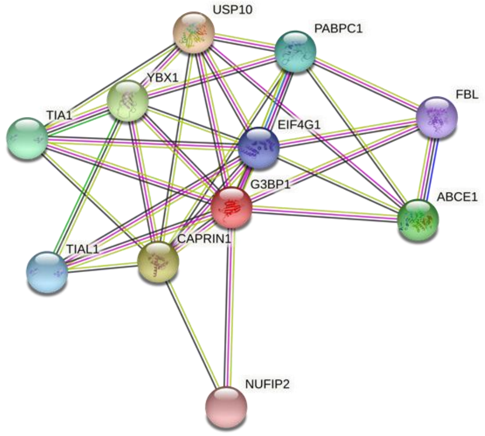

Ras GTPase-activating protein-binding protein 1

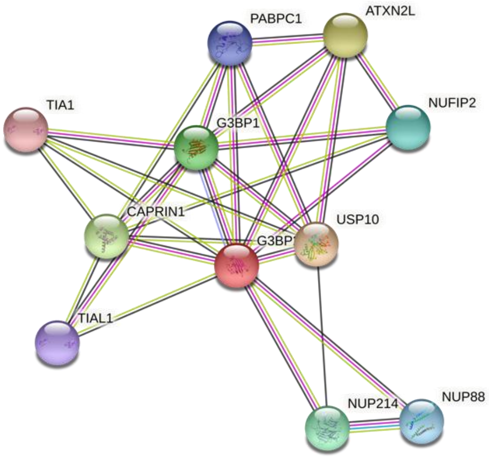

Ras GTPase-activating protein-binding protein 2

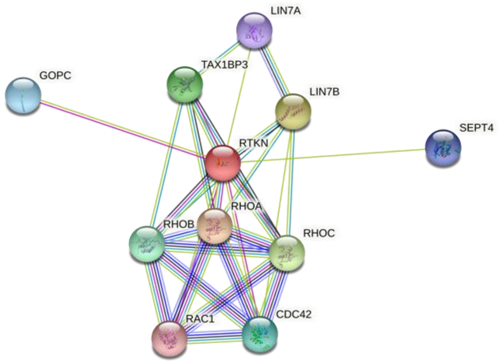

Rhotekin

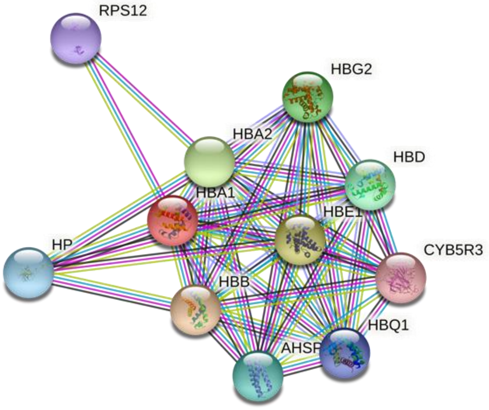

Hemoglobin subunit alpha

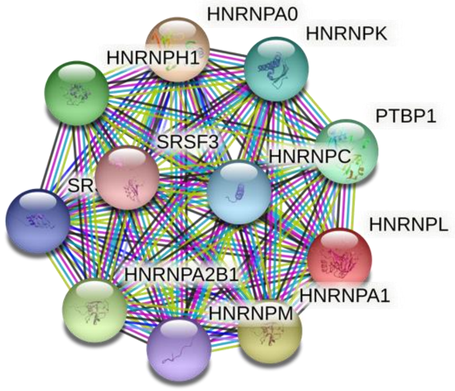

Heterogeneous nuclear ribonucleoprotein L

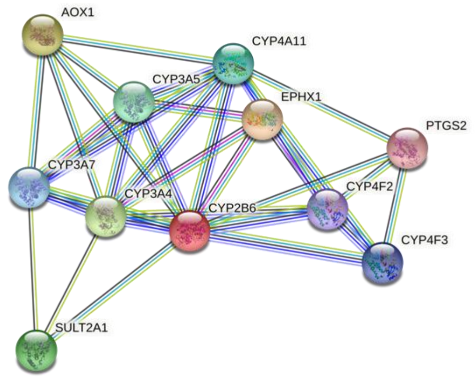

cytochrome P450-2B6

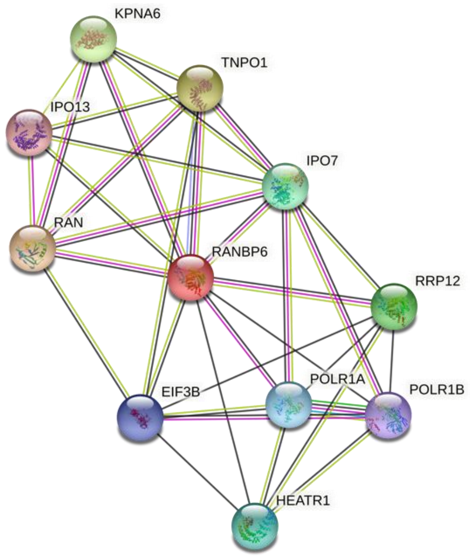

Ran-binding protein 6
