# Supplementary figures and images for: SARS-CoV-2 Proteome Harbors Peptides Which Are Able to Trigger Autoimmunity Responses: Implications for Infection, Vaccination, and Population Coverage
Source: Front Immunol. 2021 Aug 10;12:705772. doi: 10.3389/fimmu.2021.705772 (PMC8383889; doi:10.3389/fimmu.2021.705772)

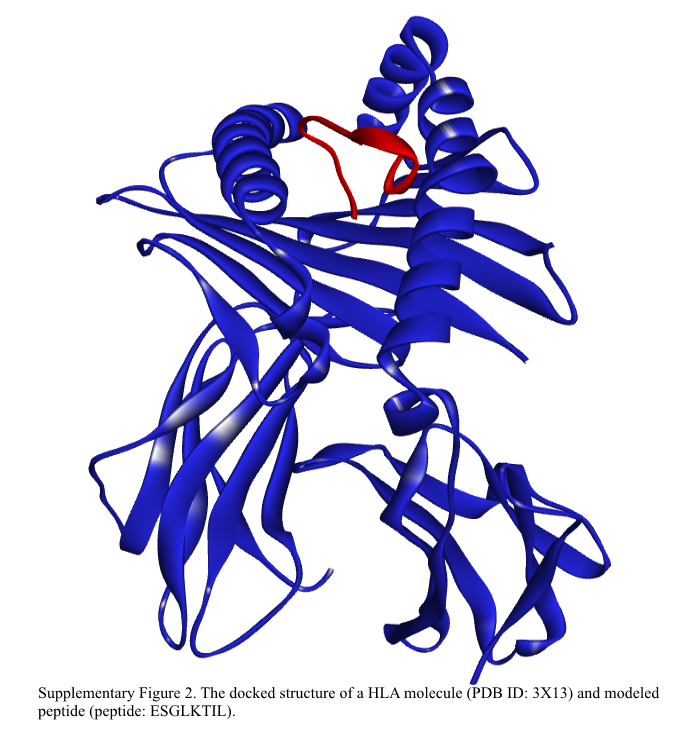

Supplement: Supplementary file 2 [file Image_2.tif]
