# Supplementary material for: SARS-CoV-2 Proteome Harbors Peptides Which Are Able to Trigger Autoimmunity Responses: Implications for Infection, Vaccination, and Population Coverage
Source: Front Immunol. 2021 Aug 10;12:705772. doi: 10.3389/fimmu.2021.705772 (PMC8383889; doi:10.3389/fimmu.2021.705772)
Supplement: Supplementary file 3 [file Table_1.docx]

Supplementary Table 1. The tissue specificity of the found human proteins. The highlighted rows are the proteins with heart specific expression.

|  | **Peptide** | **Source** | **Protein ID** | **Protein Name** | **Tissue** |
| --- | --- | --- | --- | --- | --- |
| 1 | ESGLKTIL | ORF1ab polyprotein | ANXA7 | Annexin A7 | Isoform 1 is expressed in brain, heart and skeletal muscle. Isoform 2 is more abundant in liver, lung, kidney, spleen, fibroblasts and placenta. |
| 2 | EVEKGVLP | ORF1ab polyprotein | NDST1 | Bifunctional heparan sulfate N-deacetylase/N-sulfotransferase 1 | Widely expressed. Expression is most abundant in heart, liver and pancreas. |
| 3 | DEDEEEGD | ORF1ab polyprotein | GMCL1 | Germ cell-less protein-like 1 | Low tissue specificity |
| 4 | PDEDEEEG | ORF1ab polyprotein | C2D1A | Coiled-coil and C2 domain-containing protein 1A | Low tissue specificity |
| 5 | DIQLLKSA | ORF1ab polyprotein | EMAL1 | Echinoderm microtubule-associated protein-like 1 | Ubiquitous; expressed in most tissues with the exception of thymus and peripheral blood lymphocytes |
| 6 | EVLLAPLL | ORF1ab polyprotein | AR6P4 | ADP-ribosylation factor-like protein 6-interacting protein 4 | Isoforms 3 and 7 were identified in brain, pancreas, prostate, and testis, but little or no message could be detected in other tissues. |
| 7 | YNYEPLTQ | ORF1ab polyprotein | MCM8 | DNA helicase MCM8 | Highest levels in placenta, lung and pancreas. Low levels in skeletal muscle and kidney. Expressed in various tumors with highest levels in colon and lung cancers. |
| 8 | RRSFYVYA | ORF1ab polyprotein | TPRA1 | Transmembrane protein adipocyte-associated 1 | Ubiquitous, with higher levels in heart, placenta and kidney. |
| 9 | AKKNNLPF | ORF1ab polyprotein | LGAT1 | Acyl-CoA: lysophosphatidyl glycerol acyltransferase 1 | Highly expressed in liver and placenta. Also expressed in peripheral blood, lung, kidney and brain. Detected at lower levels in colon. |
| 10 | DTSLSGFK | ORF1ab polyprotein | S12A7 | Solute carrier family 12 member 7 | Detected in muscle, brain, lung, heart and kidney. |
| 11 | SLKELLQN | ORF1ab polyprotein | CENPI | Centromere protein I | Low tissue specificity: Tissue enhanced (lymphoid) |
| 12 | PGSGVPVV | ORF1ab polyprotein | AMD | Peptidyl-glycine alpha-amidating monooxygenase precursor | Low tissue specificity: Tissue enhanced (heart) |
| 13 | RYPANSIV | ORF1ab polyprotein | BRI3 | Brain protein I3 | Low tissue specificity |
| 14 | GPPGTGKS | ORF1ab polyprotein | VPS4B | Vacuolar protein sorting-associated protein 4B | Low tissue specificity |
|  |  |  | SETX | Probable helicase senataxin | Highly expressed in skeletal muscle. Expressed in heart, fibroblast, placenta and liver. Weakly expressed in brain and lung. Expressed in the cortex of the kidney (highly expressed in tubular epithelial cells but low expression in the glomerulus). |
|  |  |  | VPS4A | Vacuolar protein sorting-associated protein 4A | Low tissue specificity |
| 15 | NVAITRAK | ORF1ab polyprotein | DNA2 | DNA replication ATP-dependent helicase/nuclease DNA2 | Low tissue specificity: Tissue enhanced (lymphoid) |
| 16 | QGPPGTGK | ORF1ab polyprotein | RENT1 | Regulator of nonsense transcripts 1 | Low tissue specificity |
|  |  |  | HELZ2 | Helicase with zinc finger domain 2 | Expressed in various tissues including heart, pancreas, skeletal muscle, colon, spleen, liver, kidney, lung, peripheral blood and placenta. |
|  |  |  | ZNFX1 | NFX1-type zinc finger-containing protein 1 | Expressed in germs cells (PubMed: 29775580, PubMed: 29769721). Not expressed in somatic tissues (PubMed: 29769721). |
| 17 | RFNVAITR | ORF1ab polyprotein | M10L1 | RNA helicase Mov10l1 | Isoform 1: Specifically expressed in testis (PubMed: 12754203). Isoform 1: In testis, present in pachytene spermatocytes but absent in postmeiotic spermatids (at protein level) (PubMed: 20534472, PubMed: 20547853). Isoform 2: Present in cardiomyocytes (at protein level) (PubMed: 11279525). Isoform 2: Heart specific (PubMed: 11854500). Isoform 3: Heart specific and is specifically expressed in cardiac myocytes (PubMed: 12754203). |
| 18 | VTLIGEAV | ORF1ab polyprotein | 6PGD | 6-phosphogluconate dehydrogenase, decarboxylating | Low tissue specificity: Tissue enhanced (blood, esophagus) |
| 19 | LALITLAT | nonstructural protein NS7a | 5HT1B | 5-hydroxytryptamine receptor 1B | Detected in cerebral artery smooth muscle cells (at protein level). Detected in brain cortex, striatum, amygdala, medulla, hippocampus, caudate nucleus and putamen. |
| 20 | DEDDSEPV | Surface glycoprotein | MYO16 | Unconventional myosin-XVI | Low tissue specificity: Tissue enhanced (brain) |
| 21 | RRARSVAS | Surface glycoprotein | SCNNA | Amiloride-sensitive sodium channel subunit alpha | Expressed in the female reproductive tract, from the fimbrial end of the fallopian tube to the endometrium (at protein level) (PubMed: 22207244). Expressed in kidney (at protein level). In the respiratory tract, expressed in the bronchial epithelium (at protein level). Highly expressed in lung. Detected at intermediate levels in pancreas and liver, and at low levels in heart and placenta (PubMed: 22207244). In skin, expressed in keratinocytes, melanocytes and Merkel cells of the epidermal sub-layers, stratum basale, stratum spinosum and stratum granulosum (at protein level) (PubMed: 28130590). Expressed in the outer root sheath of the hair follicles (at protein level) (PubMed: 28130590). Detected in both peripheral and central cells of the sebaceous gland (at protein level) (PubMed: 28130590). Expressed by eccrine sweat glands (at protein level) (PubMed: 28130590). In skin, also expressed by arrector pili muscle cells and intradermal adipocytes (PubMed: 28130590). Isoform 1 and isoform 2 predominate in all tissues. Expression of isoform 3, isoform 4 and isoform 5 is very low or not detectable, except in lung and heart (PubMed:9575806) |
| 22 | VFLLVTLA | Envelope protein | FUT10 | Alpha-(1,3)-fucosyltransferase 10 | Low tissue specificity |
| 23 | VNSVLLFL | Envelope protein | RNBP6 | Ran-binding protein 6 | Low tissue specificity |
