# Supplementary material for: SARS-CoV-2 Proteome Harbors Peptides Which Are Able to Trigger Autoimmunity Responses: Implications for Infection, Vaccination, and Population Coverage
Source: Front Immunol. 2021 Aug 10;12:705772. doi: 10.3389/fimmu.2021.705772 (PMC8383889; doi:10.3389/fimmu.2021.705772)
Supplement: Supplementary file 4 [file Table_2.docx]

Supplementary Table 2. Immunological properties of experimentally confirmed IEDB epitopes.

|  | **SARS-Cov-2 peptide** | **Related protein** | **IEDB peptide sequence** | **organism** | **Experimental HLAs for IEDB peptide** | **Disease** |
| --- | --- | --- | --- | --- | --- | --- |
|  | ESGLKTIL | trifunctional enzyme subunit alpha, mitochondrial precursor | GLKTILKDATLTALDRG | Homo sapiens | - | no immunization |
|  |  | Trifunctional enzyme subunit alpha, mitochondrial | SVDKGLKTILKDATLT | Homo sapiens | - | no immunization |
|  |  | 78 kDa gastrin-binding protein | SVDKGLKTILKDATLT | Homo sapiens | HLA-DR | respiratory system disease (chronic) |
|  |  | Annexin A7 | VESGLKTIL | Homo sapiens | HLA-B*40:01 | no immunization |
|  |  | Trifunctional enzyme subunit alpha, mitochondrial | GLKTILKDATLTALDR | Homo sapiens | HLA-DRB1*04:01 | no immunization |
|  |  | phosphoglycolate phosphatase | ATCGLKTILTL | Homo sapiens | HLA-B*57:01 | no immunization |
|  |  | Trifunctional enzyme subunit alpha, mitochondrial | AGIAQVSVDKGLKTILK | Homo sapiens | HLA-DRB1*0301, HLA-DRB3*0101 | no immunization |
|  |  | Long-chain-fatty-acid--CoA ligase 5 | ESGLKTFEQVKAIFLHPEPF | Homo sapiens | - | no immunization |
|  |  | Long-chain-fatty-acid--CoA ligase 5 | ESGLKTFEQVKAIFLHPEPFS | Homo sapiens | - | no immunization |
|  |  | annexin A7 isoform 4 | VESGLKTIL | Homo sapiens | HLA-B*40:02 | no immunization |
|  |  | phosphoglycolate phosphatase | LLGATCGLKTILTLTG | Homo sapiens | HLA-F*01:03 | no immunization |
|  |  | Trifunctional enzyme subunit alpha, mitochondrial | GLKTILKDATLTALDRGQQ | Homo sapiens | - | no immunization |
|  |  | Trifunctional enzyme subunit alpha, mitochondrial | KGLKTILKDATLTALDRGQQ | Homo sapiens | - | no immunization |
|  |  | Trifunctional enzyme subunit alpha, mitochondrial | VSVDKGLKTILKDATLTALDRG | Homo sapiens | - | no immunization |
|  |  | ***Replicase polyprotein 1ab*** | ***NESGLKTIL*** | ***SARS-CoV2*** | ***HLA-B*40:01*** | ***no immunization*** |
| **2** | EVEKGVLP | ***Replicase polyprotein 1ab*** | ***GLVEVEKGV*** | ***SARS-CoV2*** | ***HLA-A*02:01, IFNg release,*** | ***no immunization*** |
| **3** | DEDEEEGD | Acidic leucine-rich nuclear phosphoprotein 32 family member B | GLDEEDEDEDEDEEEEEGGK | Homo sapiens | HLA-DRB1*0101 | no immunization |
|  |  | NSFL1 cofactor p47 | IHDQDEDEEEEEGQRFY | Homo sapiens | - | no immunization |
|  |  | Acidic leucine-rich nuclear phosphoprotein 32 family member A | VEGLDDEEEDEDEEEY | Homo sapiens | - | no immunization |
|  |  | RNA-binding protein 28 | DDEDEEEENIESKVTKPV | Homo sapiens | - | no immunization |
|  |  | RNA-binding protein 28 | DDEDEEEENIESKVTKPVQ | Homo sapiens | - | no immunization |
|  |  | RNA-binding protein 28 | EENDDDDDDDDEEDGVFDDEDEEEE | Homo sapiens | - | no immunization |
|  |  | NSFL1 cofactor p47 | IHDQDEDEEEEEGQRF | Homo sapiens | - | no immunization |
|  |  | Nucleolin   \|  \| \| --- \| | KAKNVAEDEDEEE | Homo sapiens | - | no immunization |
|  |  | Huntingtin-associated protein 1 | LQLYSDSDEEDEDEEEEEEEKEAEE | Homo sapiens | - | no immunization |
|  |  | NSFL1 cofactor p47 | RDLIHDQDEDEEEEEGQRF | Homo sapiens | - | no immunization |
|  |  | NSFL1 cofactor p47 | RDLIHDQDEDEEEEEGQRFY | Homo sapiens | - | no immunization |
|  |  | Acidic leucine-rich nuclear phosphoprotein 32 family member B | GLDEEDEDEDEDEEEEEGGKGEKR | Homo sapiens | - | no immunization |
|  |  | Acidic leucine-rich nuclear phosphoprotein 32 family member B | GLDEEDEDEDEDEEEEEGGKGEKRK | Homo sapiens | - | no immunization |
|  |  | NSFL1 cofactor p47 | IHDQDEDEEEEEGQR | Homo sapiens | - | no immunization |
|  |  | Acidic leucine-rich nuclear phosphoprotein 32 family member B | GLDEEDEDEDEDEEEEEGGKGEK | Homo sapiens | - | no immunization |
| **4** | PDEDEEEG | RNA-binding protein 33 | ETEFPDEDEETRLY | Homo sapiens | HLA-A*01:01 | no immunization |
|  |  | NSFL1 cofactor p47 | IHDQDEDEEEEEGQRFY | Homo sapiens | - | no immunization |
|  |  | Acidic leucine-rich nuclear phosphoprotein 32 family member A | VEGLDDEEEDEDEEEY | Homo sapiens | - | no immunization |
|  |  | high mobility group protein B2 [Homo sapiens] | EDEDEEEEDEDE | Homo sapiens | - | rheumatoid arthritis(chronic) |
|  |  | HMGB1 [Homo sapiens] | EDEEDEDEEEDD | Homo sapiens | - | rheumatoid arthritis(chronic) |
|  |  | RNA-binding protein 28 | DDEDEEEENIESKVTKPV | Homo sapiens | - | no immunization |
|  |  | RNA-binding protein 28 | DDEDEEEENIESKVTKPVQ | Homo sapiens | - | no immunization |
|  |  | RNA-binding protein 28 | EENDDDDDDDDEEDGVFDDEDEEEE | Homo sapiens | - | no immunization |
|  |  | NSFL1 cofactor p47 | IHDQDEDEEEEEGQRF | Homo sapiens | - | no immunization |
|  |  | Nucleolin | KAKNVAEDEDEEE | Homo sapiens | - | no immunization |
|  |  | Huntingtin-associated protein 1 | LQLYSDSDEEDEDEEEEEEEKEAEE | Homo sapiens | - | no immunization |
|  |  | NSFL1 cofactor p47 | RDLIHDQDEDEEEEEGQRF | Homo sapiens | - | no immunization |
|  |  | NSFL1 cofactor p47 | RDLIHDQDEDEEEEEGQRFY | Homo sapiens | - | no immunization |
|  |  | Acidic leucine-rich nuclear phosphoprotein 32 family member B | GLDEEDEDEDEDEEEEEGGKGEKR | Homo sapiens | - | no immunization |
|  |  | Acidic leucine-rich nuclear phosphoprotein 32 family member B | GLDEEDEDEDEDEEEEEGGKGEKRK | Homo sapiens | - | no immunization |
|  |  | NSFL1 cofactor p47 | IHDQDEDEEEEEGQR | Homo sapiens | - | no immunization |
|  |  | RNA-binding protein 33 | TEFPDEDEETRLY | Homo sapiens | HLA-A*01:01 | no immunization |
|  |  | HMG2B, partial | KNEPEDEEEEEEEEDEDEEEEDED | Homo sapiens | HLA-DR | Lyme disease (chronic) |
|  |  | Acidic leucine-rich nuclear phosphoprotein 32 family member B | GLDEEDEDEDEDEEEEEGGK | Homo sapiens | HLA-DRB1*0101 | no immunization |
|  |  | Acidic leucine-rich nuclear phosphoprotein 32 family member B | GLDEEDEDEDEDEEEEEGGKGEK | Homo sapiens | HLA-DRB1*01:01 | no immunization |
| **5** | DIQLLKSA | E3 ubiquitin-protein ligase NRDP1 | KRDIQLLK | Homo sapiens | - | no immunization |
|  |  | Serine/threonine-protein phosphatase 2A 56 kDa regulatory subunit delta isoform | DIQLLKRTV | Homo sapiens | HLA-A*33:01 | no immunization |
|  |  | ***Replicase polyprotein 1ab*** | *GEDIQLLKA* | ***SARS-CoV1*** | *HLA-B*18:01, HLA-B*40:02, HLA-B*44:03, HLA-B*45:01* | ***no immunization*** |
| **6** | EVLLAPLL | 25-hydroxyvitamin D-1 alpha hydroxylase, mitochondrial | RSLLAPLLL | Homo sapiens | HLA-C*05:01 | no immunization |
|  |  | unknown protein eluted from human MHC allele | TIHLLAPLL | Unidentified | HLA-C*08:01 | no immunization |
|  |  | Hemojuvelin | LLAPLLSGL | Homo sapiens | HLA-A*02:01 | no immunization |
|  |  | Neurogenic locus notch homolog protein 1 | MPPLLAPLL | Homo sapiens | HLA-A*01:01 | no immunization |
|  |  | Neurogenic locus notch homolog protein 1 | MPPLLAPLL | Homo sapiens | HLA-A*01:01 | Ovarian cancer  (Chronic) |
| **7** | YNYEPLTQ | MCM8 protein | RVYNYEPLTQLK | Homo sapiens | HLA-A*03:01 | no immunization |
|  |  | DNA helicase MCM8 | RVYNYEPLTQLK | Homo sapiens | HLA-A*03:01 | no immunization |
| **8** | RRSFYVYA | ***Replicase polyprotein 1a*** | ***GMKRSFYVY*** | ***SARS-CoV1*** | ***HLA-B*15:01, HLA-A*03:01, HLA-A*29:02, HLA-A*30:02, HLA-A*31:01, HLA-B*27:05*** | ***no immunization*** |
|  |  | ***Replicase polyprotein 1ab*** | ***SFYVYANGGR*** | ***SARS-CoV1*** | ***HLA-A*03:01, HLA-A*11:01, HLA-A*31:01, HLA-A*33:01, HLA-A*68:01*** | ***no immunization*** |
| **9** | SLKELLQN | DNA polymerase zeta catalytic subunit | AVLKELLQK | Homo sapiens | HLA-A*11:01 | no immunization |
|  |  | DNA polymerase zeta catalytic subunit variant 1 | AVLKELLQK | Homo sapiens | HLA-A*11:01 | no immunization |
|  |  | Centromere protein I | QSLKELLQNW | Homo sapiens | HLA-B*57:03; HLA-B*57:01; HLA-B*58:01 | no immunization |
|  |  | S100 calcium binding protein A1 isoform 2 | KELKELLQTEL | Homo sapiens | - | skin melanoma |
|  |  | Endoribonuclease LACTB2 | DLYDYMNSLKELLK | Homo sapiens | - | no immunization |
|  |  | ***IAVLDMCAALKELLQ*** | ***Replicase polyprotein 1ab*** | ***SARS-CoV1*** | ***HLA-B*57:01, HLA-B*57:03, HLA-B*58:01*** | ***no immunization*** |
|  |  | ***LKELLQNGMNGRTIL*** | ***Replicase polyprotein 1ab*** | ***SARS-CoV1*** | ***HLA-DRB1*01:01*** | ***no immunization*** |
| **10** | PGSGVPVV | peptidylglycine alpha-amidating monooxygenase | KEPGSGVPVVL | Homo sapiens | HLA-B*40:02 | no immunization |
|  |  | Peptidyl-glycine alpha-amidating monooxygenase | GSGVPVVL | Homo sapiens | HLA-C*12:02 | no immunization |
| **11** | RYPANSIV | Brain protein I3 | YPANSIVVV | Homo sapiens | HLA-B*51:01; HLA-B*54:01 | no immunization |
|  |  | brain protein I3 isoform X4 [Homo sapiens] | YPANSIVVV | Homo sapiens | - | brain cancer(chronic) |
|  |  | brain protein I3 isoform X4 [Homo sapiens] | YPANSIVVV | Homo sapiens | HLA-B*51:01 | cervical cancer(Acute/Recent onset ) |
|  |  | Brain protein I3 | YPANSIVVV | Homo sapiens | - | breast cancer (Acute/Recent onset ) |
|  |  | Brain protein I3 | YPANSIVVV | Homo sapiens | HLA-B*51:08 | Behcet's disease(chronic) |
|  |  | Brain protein I3 | YPANSIVVV | Homo sapiens | - | skin melanoma (chronic) |
|  |  | Brain protein I3 | YPANSIVVV | Homo sapiens | - | Cancer (chronic) |
|  |  | Brain protein I3 | YPANSIVVV | Homo sapiens | HLA-B*51:01 | no immunization |
|  |  | brain protein I3 isoform X4 [Homo sapiens] | YPANSIVV | Homo sapiens | HLA-B*51:01 | cervical cancer (Acute/Recent onset ) |
|  |  | Brain protein I3 | YPANSIVV | Homo sapiens | HLA-B*51:01 | no immunization |
|  |  | Brain protein I3 | TVTRYPANSIVVVG | Homo sapiens | HLA-DPA1*02:01/DPB1*10:01 | no immunization |
|  |  | Brain protein I3 | HSRTVTRYPANSIVV | Homo sapiens | HLA-DRB5*02:02 | no immunization |
|  |  | Brain protein I3 | HSRTVTRYPANSIVVV | Homo sapiens | HLA-DRB1*15:01 | no immunization |
|  |  | Brain protein I3 | HSRTVTRYPANSIVVVG | Homo sapiens | HLA-DRB1*15:01 | no immunization |
|  |  | Brain protein I3 | SRTVTRYPANSIVV | Homo sapiens | HLA-DRB5*02:02 | no immunization |
| **12** | GPPGTGKS | ATP-dependent zinc metalloprotease YME1L1 isoform 4 | LVGPPGTGK | Homo sapiens | HLA-A*03:01 | no immunization |
|  |  | ATP-dependent metalloprotease FtsH1 homolog | LVGPPGTGK | Homo sapiens | - | no immunization |
|  |  | ATP-dependent zinc metalloprotease YME1L1 | LVGPPGTGK | Homo sapiens | HLA-A*03:01; HLA-A*34:02 | no immunization |
|  |  | YME1-like 1 (S. cerevisiae) | LVGPPGTGK | Homo sapiens | HLA-A*03:01 | no immunization |
|  |  | putative ATPases [Homo sapiens] | LVGPPGTGK  123-131 | Homo sapiens | - | nervous system cancer (chronic) |
|  |  | Regulator of nonsense transcripts 1 | IQGPPGTGK  501-509 | Homo sapiens | - | no immunization |
|  |  | NFX1-type zinc finger-containing protein 1 | IQGPPGTGK | Homo sapiens | - | no immunization |
|  |  | helicase with zinc finger domain 2 isoform 1 | IQGPPGTGK | Homo sapiens | - | no immunization |
|  |  | helicase with zinc finger domain 2 isoform 1 | IQGPPGTGK | Homo sapiens | - | skin melanoma (chronic) |
|  |  | NFX1-type zinc finger-containing protein 1 | IQGPPGTGK | Homo sapiens | - | nervous system cancer (chronic) |
|  |  | Transitional endoplasmic reticulum ATPase | YGPPGTGKTL | Homo sapiens | H2-Kq; H2-Dq | no immunization |
|  |  | ATPase family AAA domain-containing protein 3A | YGPPGTGKTL | Homo sapiens | HLA-C*01:02 | no immunization |
|  |  | Peroxisome biogenesis factor 1 | YGPPGTGKTL | Homo sapiens | HLA-B*35:03; HLA-C*07:04; HLA-C*03:03 ; HLA-B*18:01;HLA-C*01:02 | no immunization |
|  |  | ATPase family AAA domain-containing protein 2 | YGPPGTGKTL | Homo sapiens | - | no immunization |
|  |  | Transitional endoplasmic reticulum ATPase | GPPGTGKTLI | Homo sapiens | HLA-G*01:01; HLA-A*02:01; HLA-C*03:03 | no immunization |
|  |  | Fidgetin-like protein 1 | GPPGTGKTLI | Homo sapiens | - | glioblastoma |
|  |  | Vacuolar protein sorting-associated protein 4A | GPPGTGKSYLAKAVATEAN | Homo sapiens | - | no immunization |
|  |  | SKD1 protein | GPPGTGKSYLAKAVATEAN | Homo sapiens | HLA-DRA*01:01/DRB1*08:01 | no immunization |
|  |  | paraplegin-like protein | LTGPPGTGK | Homo sapiens | - | skin melanoma (chronic) |
|  |  | AFG3-like protein 2 | LTGPPGTGK | Homo sapiens | HLA-A*34:02 | no immunization |
|  |  | Replication factor C subunit 2 | IIAGPPGTGK | Homo sapiens | - | no immunization |
|  |  | tat binding protein-1 (tbp-1) | LMYGPPGTGK | Homo sapiens | - | no immunization |
|  |  | 26S protease regulatory subunit 4 | ILYGPPGTGK | Homo sapiens | HLA-A*03:01 ; HLA-A*34:02; HLA-A*11:01 | no immunization |
|  |  | proteasome (prosome, macropain) 26S subunit, ATPase, 1 | ILYGPPGTGK | Homo sapiens | HLA-A3; HLA-A*03:01 | no immunization |
|  |  | 26S protease regulatory subunit 4 | ILYGPPGTGK | Homo sapiens | - | bile duct adenocarcinoma (Metastatic) |
|  |  | proteasome (prosome, macropain) 26S subunit, ATPase, 1 | ILYGPPGTGK | Homo sapiens | - | breast cancer(Acute/Recent onset) |
|  |  | 26S protease regulatory subunit 4 | ILYGPPGTGK | Homo sapiens | - | Glioblastoma |
|  |  | 26S protease regulatory subunit 4 | ILYGPPGTGK | Homo sapiens | - | Melanoma |
|  |  | replication factor C subunit 5 isoform 4 | LLYGPPGTGK | Homo sapiens | - | skin melanoma (Acute/Recent onset ) |
|  |  | ATPase; strong similarity to peroxisome biosynthesis protein PAS1 | LLYGPPGTGK | Homo sapiens | - | breast cancer (Acute/Recent onset ) |
|  |  | thyroid receptor interactor [Homo sapiens] | LLYGPPGTGK | Homo sapiens | - | brain cancer ( chronic) |
|  |  | Peroxisome biogenesis factor 1 | LLYGPPGTGK | Homo sapiens | - | no immunization |
|  |  | 26S protease regulatory subunit 10B | LLYGPPGTGK | Homo sapiens | - | no immunization |
|  |  | ATPase; strong similarity to peroxisome biosynthesis protein PAS1 | LLYGPPGTGK | Homo sapiens | HLA-A*03:01; | no immunization |
|  |  | katanin p60 ATPase-containing subunit A-like 2 | LLYGPPGTGK | Homo sapiens | HLA-A*03:01 | no immunization |
|  |  | Katanin p60 ATPase-containing subunit A-like 2 | LLYGPPGTGK | Homo sapiens | HLA-A*03:01 | no immunization |
|  |  | Transitional endoplasmic reticulum ATPase | LLYGPPGTGK | Homo sapiens | - | no immunization |
|  |  | Peroxisome biogenesis factor 1 | LLYGPPGTGK | Homo sapiens | - | lymphoplasmacytic lymphoma |
|  |  | ruvB-like 1 | GPPGTGKTAL | Homo sapiens | HLA-B*27:05; HLA-B*07:02 | no immunization |
|  |  | ruvB-like 1 | GPPGTGKTAL | Homo sapiens | HLA-B*27:05 | experimental arthritis (Acute/Recent onset ) |
|  |  | ruvB-like 1 | GPPGTGKTAL | Homo sapiens | - | skin melanoma(Acute/Recent onset ) |
|  |  | ruvB-like 1 isoform 3 | GPPGTGKTAL | Homo sapiens | - | skin melanoma (chronic) |
|  |  | 26S protease regulatory subunit 6A | LMYGPPGTGK | Homo sapiens | - | no immunization |
|  |  | ATPase family AAA domain-containing protein 3A | LMYGPPGTGK | Homo sapiens | HLA-A3 | no immunization |
|  |  | tat binding protein-1 (tbp-1) | LMYGPPGTGK | Homo sapiens | - | no immunization |
|  |  | Katanin p60 ATPase-containing subunit A1 | MVGPPGTGK | Homo sapiens | - | no immunization |
|  |  | katanin p60 ATPase-containing subunit A1 isoform 2 | MVGPPGTGK | Homo sapiens | - | no immunization |
|  |  | Katanin p60 ATPase-containing subunit A-like 1 | MVGPPGTGK | Homo sapiens | - | no immunization |
|  |  | Helicase with zinc finger domain 2 | GPPGTGKTI | Homo sapiens | HLA-C*01:02; HLA-B*42:01 | no immunization |
|  |  | Transitional endoplasmic reticulum ATPase | GPPGTGKTLIARAVANETG | Homo sapiens | HLA-DQA1*05:05/DQB1*03:01 | no immunization |
|  |  | valosin-containing protein | GPPGTGKTLIARAVANETG | Homo sapiens | HLA-DRA*01:01/DRB1*08:01 | no immunization |
|  |  | MOV10 protein | IIFGPPGTGK | Homo sapiens | HLA-A*03:01 | no immunization |
|  |  | Helicase MOV-10 | IIFGPPGTGK | Homo sapiens | HLA-A*03:01 | no immunization |
|  |  | Katanin p60 ATPase-containing subunit A1 | GPPGTGKTL | Homo sapiens | HLA-C*01:02 | no immunization |
|  |  | peroxisome biogenesis factor 6 isoform 2 | GPPGTGKTL | Homo sapiens | - | brain cancer(chronic) |
|  |  | Katanin p60 ATPase-containing subunit A1 | GPPGTGKTLL | Homo sapiens | HLA-C*01:02 | no immunization |
|  |  | Peroxisome biogenesis factor 1 | GPPGTGKTLL | Homo sapiens | HLA-A*02:01 | no immunization |
|  |  | Katanin p60 ATPase-containing subunit A1 | VGPPGTGKTL | Homo sapiens | HLA-C*01:02 | no immunization |
|  |  | fidgetin-like protein 1 isoform 1 | LLFGPPGTGK | Homo sapiens | HLA-A*03:01 | no immunization |
|  |  | Transitional endoplasmic reticulum ATPase | GILLYGPPGTGK | Homo sapiens | HLA-C*12:02 | no immunization |
|  |  | Peroxisome biogenesis factor 1 | GILLYGPPGTGK | Homo sapiens | HLA-A*11:01 | no immunization |
|  |  | valosin-containing protein | GPPGTGKTLIARAVANETGA | Homo sapiens | HLA-DRA*01:01/DRB1*08:01 | no immunization |
|  |  | Transitional endoplasmic reticulum ATPase | GPPGTGKTLIARAVANETGAF | Homo sapiens | HLA-DQA1*05:05/DQB1*03:01 | no immunization |
|  |  | AQR | VVGPPGTGK | Homo sapiens | - | no immunization |
|  |  | RNA helicase aquarius | VVGPPGTGK | Homo sapiens | - | chronic skin melanoma |
|  |  | valosin-containing protein | LLYGPPGTGKTLIARAV | Homo sapiens | HLA-DR | respiratory system disease(chronic) |
|  |  | similar to 26S proteasome subunit p45 | LLYGPPGTGKTLLARAV | Homo sapiens | HLA-DR | respiratory system disease(chronic) |
|  |  | DNA-binding protein SMUBP-2 | IIHGPPGTGK | Homo sapiens | HLA-A*03:01 | no immunization |
|  |  | Probable helicase senataxin | LIHGPPGTGK | Homo sapiens | HLA-A*03:01 | no immunization |
|  |  | Up-regulator of cell proliferation | VPGTGKST | Homo sapiens | HLA-B*07:02 | no immunization |
|  |  | 26S protease regulatory subunit 7 | FGPPGTGKTL | Homo sapiens | HLA-C*01:02 | no immunization |
|  |  | Mov10l1, Moloney leukemia virus 10-like 1, homolog (mouse) [Homo sapiens] | ILFGPPGTGK | Homo sapiens | - | no immunization |
|  |  | proteasome (prosome, macropain) 26S subunit, ATPase, 1 | ILYGPPGTGKTLLAK | Homo sapiens | - | no immunization |
|  |  | ruvB-like 1 isoform 3 | LLAGPPGTGK | Homo sapiens | - | no immunization |
|  |  | Katanin p60 ATPase-containing subunit A-like 2 | LLYGPPGTGKTLLAK | Homo sapiens | - | no immunization |
|  |  | NFX1-type zinc finger-containing protein 1 | IQGPPGTGKTY | Homo sapiens | - | no immunization |
|  |  | 26S proteasome regulatory subunit 4 | GVILYGPPGTGK | Homo sapiens | HLA-C*12:02 | no immunization |
|  |  | 26S proteasome regulatory subunit 8 | GVLLYGPPGTGK | Homo sapiens | HLA-C*12:02 | no immunization |
|  |  | ruvB-like 1 isoform 2 | RAVLLAGPPGTGKTALA | Homo sapiens | HLA-F*01:03 | no immunization |
|  |  | 26S proteasome regulatory subunit 4 | ILYGPPGTGKTL | Homo sapiens | HLA-C*03:04 | no immunization |
|  |  | 26S proteasome regulatory subunit 4 | ILYGPPGTGKTLL | Homo sapiens | HLA-A*02:02 | no immunization |
|  |  | Vacuolar protein sorting-associated protein 4B | LLFGPPGTGKSY | Homo sapiens | HLA-B*15:01 | no immunization |
|  |  | Katanin p60 ATPase-containing subunit A1 | MVGPPGTGK | Homo sapiens | HLA-A*34:02 | no immunization |
|  |  | Tensin-1 | GPPGTGFHGSTVS | Homo sapiens | - | no immunization |
|  |  | Katanin p60 ATPase-containing subunit A1 | GPPGTGKTLLAKAV | Homo sapiens | - | glioblastoma |
|  |  | NFX1-type zinc finger-containing protein 1 | QGPPGTGKTY | Homo sapiens | - | no immunization |
|  |  | Regulator of nonsense transcripts 1 | GPPGTGKTVTSATIVYH | Homo sapiens | HLA-DPA1*02:01/DPB1*14:01 | no immunization |
|  |  | GRIP and coiled-coil domain-containing protein 2 | TPGTGKSKLETLPKEDLIK | Homo sapiens | HLA-DPA1*02:01/DPB1*13:01 | no immunization |
|  |  | Vacuolar protein sorting-associated protein 4A | FGPPGTGKSYLAKAVATEANNSTF | Homo sapiens | - | no immunization |
|  |  | Vacuolar protein sorting-associated protein 4A | GPPGTGKSYLAKAVATEANNST | Homo sapiens | - | no immunization |
|  |  | Transitional endoplasmic reticulum ATPase | GPPGTGKTLIARAVANETGAFF | Homo sapiens | - | no immunization |
|  |  | Tensin-1 | MMGPPGTGF | Homo sapiens | - | no immunization |
|  |  | Transitional endoplasmic reticulum ATPase | YGPPGTGKTLIARAVANETGAF | Homo sapiens | - | no immunization |
|  |  | 26S protease regulatory subunit 8 | LLYGPPGTGK | Homo sapiens | HLA-A*03:01;HLA-B*07:02;HLA-B*35:03;HLA-C*04:01;HLA-C*07:02 | no immunization |
|  |  | Intron-binding protein aquarius | VVGPPGTGK | Homo sapiens | HLA-A*03:01 | no immunization |
|  |  | AQR [Homo sapiens] | VVGPPGTGK | Homo sapiens | - | nervous system cancer(chronic) |
|  |  | ***Replicase polyprotein 1ab*** | ***LQGPPGTGK*** | ***SARS-CoV1*** | ***HLA-A*03:01, HLA-A*11:01, HLA-A*31:01*** | ***no immunization*** |
|  |  | ***Replicase polyprotein 1ab*** | ***TLQGPPGTGK*** | ***SARS-CoV1*** | ***HLA-A*03:01, HLA-A*11:01, HLA-A*31:01, HLA-A*68:01*** | ***no immunization*** |
| **13** | NVAITRAK | hCG32858 | RRLNVAITR | Homo sapiens | - | breast cancer(Acute/Recent onset ) |
|  |  | hCG32858 | RRLNVAITR | Homo sapiens | HLA-B*27:05 | no immunization |
|  |  | DNA replication ATP-dependent helicase/nuclease DNA2 | RRLNVAITR | Homo sapiens | HLA-B*27:05 | no immunization |
|  |  | ***Replicase polyprotein 1ab*** | ***RFNVAITRAK*** | ***SARS-CoV1*** | ***HLA-A*03:01, HLA-A*11:01, HLA-A*31:01, HLA-A*68:01*** | ***no immunization*** |
|  |  | ***Replicase polyprotein 1ab*** | ***VNRFNVAITRAKIGI*** | ***SARS-CoV1*** | ***HLA-DRB1*01:01*** | ***no immunization*** |
|  |  | ***Orf1ab*** | ***NANRFNVAITRAKKG*** | ***Human coronavirus 229E*** | ***IFNg release*** | ***no immunization*** |
|  |  | ***Replicase polyprotein 1ab*** | ***NVNRFNVAITRAKKG*** | ***Betacoronavirus 1*** | ***IFNg release*** | ***no immunization*** |
|  |  | ***Replicase polyprotein 1ab*** | ***NVNRFNVAITRAKVG*** | ***SARS-CoV2*** | ***IFNg release, IL-5 release*** | ***no immunization*** |
| **14** | QGPPGTGK | ruvB-like 1 isoform 2 | RAVLLAGPPGTGKTALA | Homo sapiens | HLA-F*01:03 | no immunization |
|  |  | Transitional endoplasmic reticulum ATPase | LLYGPPGTGK | Homo sapiens | HLA-A*03:01 | no immunization |
|  |  | replication factor C subunit 5 isoform 4 | LLYGPPGTGK | Homo sapiens | - | skin melanoma(Acute/Recent onset) |
|  |  | ATPase; strong similarity to peroxisome biosynthesis protein PAS1 (PID:g1172019); coded for by human cDNA C04279 (NID:g1467530), partial | LLYGPPGTGK | Homo sapiens | - | breast cancer(Acute/Recent onset) |
|  |  | ATPase; strong similarity to peroxisome biosynthesis protein PAS1 (PID:g1172019); coded for by human cDNA C04279 (NID:g1467530), partial | LLYGPPGTGK | Homo sapiens | HLA-A*03:01 | no immunization |
|  |  | thyroid receptor interactor [Homo sapiens] | LLYGPPGTGK | Homo sapiens | - | brain cancer(chronic) |
|  |  | Peroxisome biogenesis factor 1 | LLYGPPGTGK | Homo sapiens | - | no immunization |
|  |  | Peroxisome biogenesis factor 1 | LLYGPPGTGK | Homo sapiens | - | lymphoplasmacytic lymphoma |
|  |  | 26S protease regulatory subunit 10B | LLYGPPGTGK | Homo sapiens | - | no immunization |
|  |  | 26S protease regulatory subunit 8 | LLYGPPGTGK | Homo sapiens | - | no immunization |
|  |  | katanin p60 ATPase-containing subunit A-like 2 | LLYGPPGTGK | Homo sapiens | HLA-A*03:01 | no immunization |
|  |  | 26S protease regulatory subunit 4 | ILYGPPGTGK | Homo sapiens | HLA-A*03:01; HLA-A*11:01 | no immunization |
|  |  | 26S protease regulatory subunit 4 | ILYGPPGTGK | Homo sapiens | - | bile duct adenocarcinoma(Metastatic), breast cancer(Acute/Recent onset), glioblastoma, melanoma |
|  |  | ruvB-like 1 | GPPGTGKTAL | Homo sapiens | HLA-B*27:05, HLA-B*07:02 | no immunization |
|  |  | ruvB-like 1 | GPPGTGKTAL | Homo sapiens | HLA-B*27:05 | experimental arthritis(Acute/Recent onset) |
|  |  | ruvB-like 1 | GPPGTGKTAL | Homo sapiens | - | skin melanoma(Acute/Recent onset) |
|  |  | ruvB-like 1 isoform 3 | GPPGTGKTAL | Homo sapiens | - | skin melanoma(chronic) |
|  |  | ATP-dependent metalloprotease FtsH1 homolog | LVGPPGTGK | Homo sapiens | HLA-A*03:01; HLA-A*34:02 | no immunization |
|  |  | YME1-like 1 (S. cerevisiae) | LVGPPGTGK | Homo sapiens | HLA-A*03:01 | no immunization |
|  |  | ATP-dependent zinc metalloprotease YME1L1 isoform 4 | LVGPPGTGK | Homo sapiens | HLA-A*03:01; HLA-A*34:02 | no immunization |
|  |  | ATP-dependent zinc metalloprotease YME1L1 isoform 4 | LVGPPGTGK | Homo sapiens | HLA-A*03:01 | no immunization |
|  |  | putative ATPases [Homo sapiens] | LVGPPGTGK | Homo sapiens | - | nervous system cancer(chronic) |
|  |  | Regulator of nonsense transcripts 1 | IQGPPGTGK | Homo sapiens | - | no immunization |
|  |  | NFX1-type zinc finger-containing protein 1 | IQGPPGTGK | Homo sapiens | - | no immunization |
|  |  | helicase with zinc finger domain 2 isoform 1 | IQGPPGTGK | Homo sapiens | - | no immunization |
|  |  | helicase with zinc finger domain 2 isoform 1 | IQGPPGTGK | Homo sapiens | - | skin melanoma |
|  |  | NFX1-type zinc finger-containing protein 1 | IQGPPGTGK | Homo sapiens | - | nervous system cancer(chronic) |
|  |  | Transitional endoplasmic reticulum ATPase | YGPPGTGKTL | Homo sapiens | H2-Kq; H2-Dq | no immunization |
|  |  | ATPase family AAA domain-containing protein 3A | YGPPGTGKTL | Homo sapiens | HLA-C*01:02 | no immunization |
|  |  | Peroxisome biogenesis factor 1 | YGPPGTGKTL | Homo sapiens | HLA-B*35:03; HLA-C*07:04; HLA-C*03:03; HLA-B*18:01; HLA-C*01:02 | no immunization |
|  |  | ATPase family AAA domain-containing protein 2 | YGPPGTGKTL | Homo sapiens | - | no immunization |
|  |  | 26S protease regulatory subunit 6A | LMYGPPGTGK | Homo sapiens | - | no immunization |
|  |  | ATPase family AAA domain-containing protein 3A | LMYGPPGTGK | Homo sapiens | HLA-A3 | no immunization |
|  |  | tat binding protein-1 (tbp-1) | LMYGPPGTGK | Homo sapiens | - | no immunization |
|  |  | Katanin p60 ATPase-containing subunit A1 | MVGPPGTGK | Homo sapiens | HLA-A*68:01; HLA-A*34:01; HLA-A*34:02 | no immunization |
|  |  | katanin p60 ATPase-containing subunit A1 isoform 2 | MVGPPGTGK | Homo sapiens | - | no immunization |
|  |  | Katanin p60 ATPase-containing subunit A-like 1 | MVGPPGTGK | Homo sapiens | - | no immunization |
|  |  | Helicase with zinc finger domain 2 | GPPGTGKTI | Homo sapiens | HLA-C*01:02; HLA-B*42:01 | no immunization |
|  |  | Transitional endoplasmic reticulum ATPase | GPPGTGKTLIARAVANETG | Homo sapiens | HLA-DQA1*05:05/DQB1*03:01; | no immunization |
|  |  | valosin-containing protein | GPPGTGKTLIARAVANETG | Homo sapiens | HLA-DRA*01:01/DRB1*08:01 | no immunization |
|  |  | MOV10 protein | IIFGPPGTGK | Homo sapiens | HLA-A*03:01; | no immunization |
|  |  | putative helicase MOV-10 isoform 1 | IIFGPPGTGK | Homo sapiens | HLA-A*03:01 | no immunization |
|  |  | AQR [Homo sapiens] | VVGPPGTGK | Homo sapiens | - | nervous system cancer(chronic) |
|  |  | Intron-binding protein aquarius | VVGPPGTGK | Homo sapiens | HLA-A*03:01 | no immunization |
|  |  | AQR [Homo sapiens] | VVGPPGTGK | Homo sapiens | - | no immunization |
|  |  | RNA helicase aquari**u**s | VVGPPGTGK | Homo sapiens | - | chronic skin melanoma |
|  |  | Transitional endoplasmic reticulum ATPase | GPPGTGKTLI | Homo sapiens | HLA-G*01:01; HLA-A*02:01; HLA-C*03:03 | no immunization |
|  |  | Fidgetin-like protein 1 | GPPGTGKTLI | Homo sapiens | - | Glioblastoma |
|  |  | Vacuolar protein sorting-associated protein 4A | GPPGTGKSYLAKAVATEAN | Homo sapiens | - | no immunization |
|  |  | SKD1 protein | GPPGTGKSYLAKAVATEAN | Homo sapiens | HLA-DRA*01:01/DRB1*08:01 | no immunization |
|  |  | paraplegin-like protein | LTGPPGTGK | Homo sapiens | - | chronic skin melanoma |
|  |  | AFG3-like protein 2 | LTGPPGTGK | Homo sapiens | HLA-A*34:02 | no immunization |
|  |  | Replication factor C subunit 2 | IIAGPPGTGK | Homo sapiens | - | no immunization |
|  |  | Katanin p60 ATPase-containing subunit A1 | GPPGTGKTL | Homo sapiens | HLA-C*01:02 | no immunization |
|  |  | peroxisome biogenesis factor 6 isoform 2 | GPPGTGKTL | Homo sapiens | - | chronic brain cancer |
|  |  | Katanin p60 ATPase-containing subunit A1 | GPPGTGKTLL | Homo sapiens | HLA-C*01:02 | no immunization |
|  |  | Peroxisome biogenesis factor 1 | GPPGTGKTLL | Homo sapiens | HLA-A*02:01 | no immunization |
|  |  | Katanin p60 ATPase-containing subunit A1 | VGPPGTGKTL | Homo sapiens | HLA-C*01:02 | no immunization |
|  |  | Transitional endoplasmic reticulum ATPase | GILLYGPPGTGK | Homo sapiens | HLA-C*12:02 | no immunization |
|  |  | Peroxisome biogenesis factor 1 | GILLYGPPGTGK | Homo sapiens | HLA-A*11:01 | no immunization |
|  |  | valosin-containing protein | GPPGTGKTLIARAVANETGA | Homo sapiens | HLA-DRA*01:01/DRB1*08:01 | no immunization |
|  |  | Transitional endoplasmic reticulum ATPase | GPPGTGKTLIARAVANETGAF | Homo sapiens | HLA-DQA1*05:05/DQB1*03:01 | no immunization |
|  |  | valosin-containing protein | LLYGPPGTGKTLIARAV | Homo sapiens | HLA-DR | respiratory system disease (chronic) |
|  |  | similar to 26S proteasome subunit p45 | LLYGPPGTGKTLLARAV | Homo sapiens | HLA-DR | respiratory system disease (chronic) |
|  |  | 26S protease regulatory subunit 7 | FGPPGTGKTL | Homo sapiens | HLA-C*01:02 | no immunization |
|  |  | Mov10l1, Moloney leukemia virus 10-like 1, homolog (mouse) [Homo sapiens] | ILFGPPGTGK | Homo sapiens | - | no immunization |
|  |  | proteasome (prosome, macropain) 26S subunit, ATPase, 1 | ILYGPPGTGKTLLAK | Homo sapiens | - | no immunization |
|  |  | ruvB-like 1 isoform 3 | LLAGPPGTGK | Homo sapiens | - | no immunization |
|  |  | Katanin p60 ATPase-containing subunit A-like 2 | LLYGPPGTGKTLLAK | Homo sapiens | - | no immunization |
|  |  | NFX1-type zinc finger-containing protein 1 | IQGPPGTGKTY | Homo sapiens | - | no immunization |
|  |  | 26S proteasome regulatory subunit 4 | GVILYGPPGTGK | Homo sapiens | HLA-C*12:02 | no immunization |
|  |  | 26S proteasome regulatory subunit 8 | GVLLYGPPGTGK | Homo sapiens | HLA-C*12:02 | no immunization |
|  |  | 26S proteasome regulatory subunit 4 | ILYGPPGTGKTL | Homo sapiens | HLA-C*03:04 | no immunization |
|  |  | 26S proteasome regulatory subunit 4 | ILYGPPGTGKTLL | Homo sapiens | HLA-A*02:02 | no immunization |
|  |  | Vacuolar protein sorting-associated protein 4B | LLFGPPGTGKSY | Homo sapiens | HLA-B*15:01 | no immunization |
|  |  | Tensin-1 | GPPGTGFHGSTVS | Homo sapiens | - | no immunization |
|  |  | Katanin p60 ATPase-containing subunit A1 | GPPGTGKTLLAKAV | Homo sapiens | - | glioblastoma |
|  |  | NFX1-type zinc finger-containing protein 1 | QGPPGTGKTY | Homo sapiens | - | no immunization |
|  |  | Regulator of nonsense transcripts 1 | GPPGTGKTVTSATIVYH | Homo sapiens | HLA-DPA1*02:01/DPB1*14:01 | no immunization |
|  |  | Vacuolar protein sorting-associated protein 4A | FGPPGTGKSYLAKAVATEANNSTF | Homo sapiens | - | no immunization |
|  |  | Vacuolar protein sorting-associated protein 4A | GPPGTGKSYLAKAVATEANNST | Homo sapiens | - | no immunization |
|  |  | Transitional endoplasmic reticulum ATPase | GPPGTGKTLIARAVANETGAFF | Homo sapiens | - | no immunization |
|  |  | Tensin-1 | MMGPPGTGF | Homo sapiens | - | no immunization |
|  |  | Transitional endoplasmic reticulum ATPase | YGPPGTGKTLIARAVANETGAF | Homo sapiens | - | no immunization |
|  |  | Fidgetin-like protein 1 isoform 1 | LLFGPPGTGK | Homo sapiens | HLA-A*03:01 | no immunization |
|  |  | ***Replicase polyprotein 1ab*** | ***LQGPPGTGK*** | ***SARS-CoV1*** | ***HLA-A*03:01, HLA-A*11:01, HLA-A*31:01*** | ***no immunization*** |
|  |  | ***Replicase polyprotein 1ab*** | ***TLQGPPGTGK*** | ***SARS-CoV1*** | ***HLA-A*03:01, HLA-A*11:01, HLA-A*31:01, HLA-A*68:01*** | ***no immunization*** |
| **15** | RFNVAITR | ***Replicase polyprotein 1ab*** | ***RFNVAITRAK*** | ***SARS-CoV1*** | ***HLA-A*03:01, HLA-A*11:01, HLA-A*31:01, HLA-A*68:01*** | ***no immunization*** |
|  |  | ***Replicase polyprotein 1ab*** | ***RFNVAITRAK*** | ***SARS-CoV1*** | ***HLA-DRB1*01:01*** | ***no immunization*** |
|  |  | ***Replicase polyprotein 1ab*** | ***NANRFNVAITRAKKG*** | ***Human coronavirus 229E*** | ***IFNg release*** | ***no immunization*** |
|  |  | ***Replicase polyprotein 1ab*** | ***NVNRFNVAITRAKKG*** | ***Betacoronavirus 1*** | ***IFNg release*** | ***no immunization*** |
|  |  | ***Replicase polyprotein 1ab*** | ***NVNRFNVAITRAKVG*** | ***SARS-CoV2*** | ***IFNg release, IL-5 release*** | ***no immunization*** |
| **16** | VTLIGEAV | NOP56 protein, partial | SLSALIGEAV | Homo sapiens | HLA-A*02:01 | no immunization |
|  |  | 6-phosphogluconate dehydrogenase, decarboxylating isoform 2 | VPVTLIGEAVF | Homo sapiens | HLA-B*35:01 | no immunization |
|  |  | 6-phosphogluconate dehydrogenase, decarboxylating | VPVTLIGEA | Homo sapiens | HLA-H2-Dq; HLA-B*55:01 | no immunization |
|  |  | 6-phosphogluconate dehydrogenase, decarboxylating | VPVTLIGEAVF | Homo sapiens | HLA-B*35:01; H2-Dq | no immunization |
|  |  | Nucleolar protein 56 | APSLSALIGEAVGAR | Homo sapiens | HLA-DR1 | no immunization |
|  |  | 6-phosphogluconate dehydrogenase, decarboxylating isoform 2 | GQKGTGKWTAISALEYGVPVTLIGEAVF | Homo sapiens | HLA-B*15:01 | no immunization |
|  |  | Nucleolar protein 56 | ALIGEAVGARLI | Homo sapiens | HLA-B*13:01 | no immunization |
| **17** | LALITLAT | ***ORF7a*** | ***LITLATCELYHYQECV*** | ***SARS-CoV2*** | ***HLA class I*** | ***no immunizatio*** |
|  |  | ***ORF7a*** | ***LITLATCELYHYQECVR*** | ***SARS-CoV2*** | ***IFNg release*** | ***no immunizatio*** |
|  |  | ***ORF7a*** | ***MKIILFLALITLATC*** | ***SARS-CoV2*** | ***IFNg release*** | ***no immunizatio*** |
|  |  | ***ORF7a*** | ***IILFLALITLATCEL*** | ***SARS-CoV2*** | ***IFNg release*** | ***no immunizatio*** |
| **18** | DEDDSEPV | Ras GTPase-activating protein-binding protein 1 | VVFDDSEPVQK | Homo sapiens | HLA-A*11:01 | no immunization |
|  |  | Ras GTPase-activating protein-binding protein 1 | VVFDDSEPVQK | Homo sapiens | - | melanoma |
|  |  | Ras GTPase-activating protein-binding protein 2 | VVFDDSEPVQRIL | Homo sapiens | HLA-Mamu-B*008:01,HLA-B*27:05 | no immunization |
|  |  | ras GTPase-activating protein-binding protein 2 isoform b | VVFDDSEPVQRIL | Homo sapiens | - | no immunization |
|  |  | ras GTPase-activating protein-binding protein 2 isoform b | VVFDDSEPVQR | Homo sapiens | - | no immunization |
|  |  | ras GTPase-activating protein-binding protein 2 isoform a | VVFDDSEPVQR | Homo sapiens | - | brain cancer (Chronic) |
|  |  | Ras GTPase-activating protein-binding protein 1 | VVFDDSEPV | Homo sapiens | HLA-C*08:01 | no immunization |
|  |  | ***Spike glycoprotein*** | ***DDSEPVLKGVKLHYT*** | ***SARS-CoV1*** | ***HLA-DRB1*01:01*** | ***no immunization*** |
|  |  | ***Spike glycoprotein*** | ***CKFDEDDSEPVLKGVKLHYT*** | ***SARS-CoV1*** | ***qualitative binding*** | ***no immunization*** |
|  |  | ***Spike glycoprotein*** | ***DEDDSEPVL*** | ***SARS-CoV1*** | ***HLA-B*40:01, HLA-B*40:02*** | ***no immunization*** |
|  |  | ***Spike glycoprotein*** | ***FDEDDSEPVLKGVKLHYT*** | ***SARS-CoV2*** | ***qualitative binding*** | ***no immunization*** |
|  |  | ***Spike glycoprotein*** | ***GCCSCGSCCKFDEDDSEPVL*** | ***SARS-CoV2*** | ***qualitative binding*** | ***no immunization*** |
|  |  | ***Spike glycoprotein*** | ***CCSCGSCCKFDEDDSEPVLKGVKL*** | ***SARS-CoV2*** | ***HLA-B*40:01*** | ***no immunization*** |
|  |  | ***Spike glycoprotein*** | ***FDEDDSEPVL*** | ***SARS-CoV2*** | ***qualitative binding*** | ***no immunization*** |
| **19** | RRARSVAS | Rhotekin | DHSPRARSVAP | Homo sapiens | HLA-A*66:01 | no immunization |
|  |  | ***Spike glycoprotein*** | ***SPRRARSVA*** | ***SARS-CoV2*** | ***IFNg release, qualitative binding, TNFa release*** | ***no immunization*** |
|  |  | ***Spike glycoprotein*** | ***SPRRARSV*** | ***SARS-CoV2*** | ***HLA-B*08:01*** | ***no immunization*** |
|  |  | ***Spike glycoprotein*** | ***AGCLIGAEHVNNSYECDIPIGAGICASYQTQTNSPRRARSVAS*** | ***SARS-CoV2*** | ***HLA class I*** | ***no immunization*** |
|  |  | ***Spike glycoprotein*** | ***ARSVASQSIIAYTMSLGAENSVAYSNNSIAIPTNFTISVTTEI*** | ***SARS-CoV2*** | ***HLA class I*** | ***no immunization*** |
|  |  | ***Spike glycoprotein*** | ***SYQTQTNSPRRARSVA*** | ***SARS-CoV2*** | ***HLA class I*** | ***no immunization*** |
|  |  | ***Spike glycoprotein*** | ***QTQTNSPRRARSVAS*** | ***SARS-CoV2*** | ***qualitative binding, neutralization*** | ***no immunization*** |
| **20** | VFLLVTLA | Hemoglobin alpha chain | LRVDPVNFKLLSHCLLVTLA | Homo sapiens | HLA-DPA1*01:03/DPB1*04:02 | no immunization |
|  |  | Hemoglobin subunit alpha | LLVTLAAHLPAEFTPAVHASLDKF | Homo sapiens | - | no immunization |
|  |  | unknown protein eluted from human MHC allele | LLSHSLLVTLA | Homo sapiens | HLA-A*02:02; HLA-A*02:05 | no immunization |
|  |  | Hemoglobin subunit alpha | DPVNFKLLSHCLLVTLAAH | Homo sapiens | HLA-DPA1*01:03/DPB1*04:02 | no immunization |
|  |  | Hemoglobin subunit zeta | DPVNFKLLSHCLLVTLA | Homo sapiens | - | no immunization |
|  |  | Hemoglobin subunit alpha | LLVTLAAHLPA | Homo sapiens | - | no immunization |
|  |  | Hemoglobin subunit alpha | LLVTLAAHLPAE | Homo sapiens | - | no immunization |
|  |  | Hemoglobin subunit alpha | LLVTLAAHLPAEFTPAV | Homo sapiens | - | no immunization |
|  |  | Hemoglobin subunit alpha | LLVTLAAHLPAEFTPAVHASLDK | Homo sapiens | - | no immunization |
|  |  | ***Envelope small membrane protein*** | ***FLLVTLAIL*** | ***SARS-CoV1*** | ***HLA-A*02:01, HLA-A*02:02, HLA-A*02:03, HLA-A*02:06, HLA-A*68:02*** | ***no immunization*** |
|  |  | ***Envelope small membrane protein*** | ***FLLVTLAILTALRLC*** | ***Other SARS*** | ***HLA-DRB1*01:01, IFNg release, IL-5 release*** | ***no immunization*** |
|  |  | ***Envelope small membrane protein*** | ***FVVFLLVTL*** | ***SARS-CoV1*** | ***HLA-A*02:01, HLA-A*02:02, HLA-A*02:03, HLA-A*02:06, HLA-A*68:02*** | ***no immunization*** |
|  |  | ***Envelope small membrane protein*** | ***LLVTLAILTA*** | ***SARS-CoV1*** | ***HLA-A*02:01, HLA-A*02:02, HLA-A*02:03, HLA-A*02:06*** | ***no immunization*** |
|  |  | ***Envelope small membrane protein*** | ***AFVVFLLVTLAILTA*** | ***Other SARS*** | ***HLA-DRB1*01:01*** | ***no immunization*** |
|  |  | ***Envelope small membrane protein*** | ***FLAFVVFLLVTLAIL*** | ***Other SARS*** | ***HLA-DRB1*01:01, IFNg release*** | ***no immunization*** |
|  |  | ***Envelope small membrane protein*** | ***LAFVVFLLVTLAILT*** | ***Other SARS*** | ***HLA-DRB1*01:01, IFNg release, IL-5 release*** | ***no immunization*** |
|  |  | ***Envelope small membrane protein*** | ***SVLLFLAFVVFLLVT*** | ***Other SARS*** | ***HLA-DRB1*01:01, IFNg release, IL-5 release*** | ***no immunization*** |
|  |  | ***Envelope small membrane protein*** | ***VFLLVTLAILTALRL*** | ***Other SARS*** | ***HLA-DRB1*01:01, IFNg release*** | ***no immunization*** |
|  |  | ***Envelope small membrane protein*** | ***VLLFLAFVVFLLVTL*** | ***Other SARS*** | ***HLA-DRB1*01:01, IFNg release*** | ***no immunization*** |
|  |  | ***Envelope small membrane protein*** | ***VVFLLVTLAILTALR*** | ***Other SARS*** | ***HLA-DRB1*01:01*** | ***no immunization*** |
| **21** | VNSVLLFL | Heterogeneous nuclear ribonucleoprotein L | RSVNSVLLF | Homo sapiens | HLA-C*15:05; HLA-B*58:01; HLA-B*57:03; HLA-B*15:01; HLA-B*57:01 | no immunization |
|  |  | cytochrome P450-2B6 [Homo sapiens] | MELSVLLFL | Homo sapiens | HLA-A*02:01 | no immunization |
|  |  | Ran-binding protein 6 | TVNSVLLFL | Homo sapiens | HLA-C*15:02; | no immunization |
|  |  | Heterogeneous nuclear ribonucleoprotein L | STSQKISRPGDSDDSRSVNSVLL | Homo sapiens | - | no immunization |
|  |  | heterogeneous nuclear ribonucleoprotein L isoform X3 [Homo sapiens] | RSVNSVLLF | Homo sapiens | HLA-B*58:01 | no immunization |
|  |  | Heterogeneous nuclear ribonucleoprotein L | STSQKISRPGDSDDSRSVNSVLLF | Homo sapiens | - | no immunization |
|  |  | ***Envelope small membrane protein*** | ***LIVNSVLLFL*** | ***SARS-CoV1*** | ***HLA-A*02:01, HLA-A*02:02, HLA-A*02:03, HLA-A*02:06, HLA-A*68:02*** | ***no immunization*** |
|  |  | ***Envelope small membrane protein*** | ***SVLLFLAFV*** | ***SARS-CoV1*** | ***HLA-A*02:01, HLA-A*02:02, HLA-A*02:03, HLA-A*02:06, HLA-A*68:02*** | ***no immunization*** |
|  |  | ***Envelope small membrane protein*** | ***IVNSVLLFL*** | ***SARS-CoV1*** | ***HLA-A*02:01, HLA-A*02:02, HLA-A*02:03, HLA-A*02:06, HLA-A*68:02*** | ***no immunization*** |
|  |  | ***Envelope small membrane protein*** | ***SVLLFLAFVV*** | ***SARS-CoV1*** | ***HLA-A*02:01, HLA-A*02:02, HLA-A*02:03, HLA-A*02:06, HLA-A*68:02*** | ***no immunization*** |
|  |  | ***Envelope small membrane protein*** | ***GTLIVNSVLLFLAFV*** | ***Other SARS*** | ***HLA-DRB1*01:01*** | ***no immunization*** |
|  |  | ***Envelope small membrane protein*** | ***SVLLFLAFVVFLLVT*** | ***Other SARS*** | ***HLA-DRB1*01:01*** | ***no immunization*** |
|  |  | ***Envelope small membrane protein*** | ***TGTLIVNSVLLFLAF*** | ***Other SARS*** | ***HLA-DRB1*01:01, IFNg release*** | ***no immunization*** |
|  |  | ***Envelope small membrane protein*** | ***TLIVNSVLLFLAFVV*** | ***Other SARS*** | ***HLA-DRB1*01:01, IFNg release, IL-5 release*** | ***no immunization*** |
|  |  | ***Envelope small membrane protein*** | ***VNSVLLFLAFVVFLL*** | ***Other SARS*** | ***HLA-DRB1*01:01*** | ***no immunization*** |
|  |  | ***Envelope small membrane protein*** | ***SEETGTLIVNSVLLF*** | ***SARS-CoV2*** | ***IFNg release, IL-5 release*** | ***no immunization*** |
