# Supplementary material for: SARS-CoV-2 Proteome Harbors Peptides Which Are Able to Trigger Autoimmunity Responses: Implications for Infection, Vaccination, and Population Coverage
Source: Front Immunol. 2021 Aug 10;12:705772. doi: 10.3389/fimmu.2021.705772 (PMC8383889; doi:10.3389/fimmu.2021.705772)
Supplement: Supplementary file 5 [file Table_3.docx]

Supplementary Table 3. The HLA molecules with resolved structures.

|  | **SARS-CoV-2 peptides** | **Predicted HLAs for SARS-CoV-2 peptides** | **PDB ID** | **IEDB peptides** | **Experimentally determined HLAs for IEDB epitopes** | **PDB ID** |
| --- | --- | --- | --- | --- | --- | --- |
| 1 | ESGLKTIL | HLA-B*08:01 | 3X13, 3X14 | NESGLKTIL | HLA-B*40:01 | - |
|  |  |  |  | ATCGLKTILTL | HLA-B*57:01 | 3X11, 3X12, 6BXP, 6BXQ, 3UPR |
|  |  |  |  | VESGLKTIL | HLA-B*40:02 | 5IEK, 5IEH |
|  |  |  |  | SVDKGLKTILKDATLT | HLA-DR | - |
|  |  |  |  | GLKTILKDATLTALDR | HLA-DRB1*04:01 | 5JLZ, 5LAX,5NIG, 5NI9 |
|  |  |  |  | AGIAQVSVDKGLKTILK | HLA-DRB1*0301, HLA-DRB3*0101 | - |
|  |  |  |  | NESGLKTIL | HLA-B*40:01 | - |
|  |  |  |  | LLGATCGLKTILTLTG | HLA-F*01:03 | - |
| 2 | EVEKGVLP | - | - | GLVEVEKGV | HLA-A*02:01 | 4U6X, 4U6Y, 5SWQ, 7KGQ, 7KGR, 7KGO, 7KGP, 6TDQ, 5F9J, 5FA3, 5FA4,5FDW, 5ENW, 5F7D, 6TDO, 6TDP, 6O4Z, 6O4Y, 6O51 |
| 3 | DEDEEEGD | - | - | GLDEEDEDEDEDEEEEEGGK | HLA-DRB1*0101 | - |
| 4 | PDEDEEEG | - | - | ETEFPDEDEETRLY | HLA-A*01:01 | 6AT9 |
|  |  |  |  | TEFPDEDEETRLY | HLA-A*01:01 | 6AT9 |
|  |  |  |  | KNEPEDEEEEEEEEDEDEEEEDED | HLA-DR | - |
|  |  |  |  | GLDEEDEDEDEDEEEEEGGKGEK | HLA-DRB1*01:01 | - |
|  |  |  |  | GLDEEDEDEDEDEEEEEGGK | HLA-DRB1*0101 | - |
| 5 | DIQLLKSA | - | - | DIQLLKRTV | HLA-A*33:01 | - |
|  |  |  |  | GEDIQLLKA | HLA-B*18:01, HLA-B*40:02, HLA-B*44:03, HLA-B*45:01 | 4XXC, 4JQV, 6MT3 (HLA-B*18:01)  5IEK & 5IEH(HLA-B*40:02)  4JQX(HLA-B*44:03) |
| 6 | EVLLAPLL | HLA-B*51:07 | - | RSLLAPLLL | HLA-C*05:01 | - |
|  |  |  |  | TIHLLAPLL | HLA-C*08:01 | 4NT6 |
|  |  |  |  | MPPLLAPLL | HLA-A*02:01 | 4U6X |
|  |  |  |  |  | HLA-A*24:02 | 3WLB |
|  |  |  |  |  | HLA-B*35:03 | - |
|  |  |  |  |  | HLA-B*44:02 | - |
|  |  |  |  |  | HLA-C*05:01 | - |
|  |  |  |  |  | HLA-C*12:03 | - |
| 7 | YNYEPLTQ | - | - | RVYNYEPLTQLK | HLA-A*03:01 | 6O9B |
|  |  |  |  |  | HLA-B*07:02 | 6AT5 |
|  |  |  |  |  | HLA-C*07:02 | 5VGE |
| 8 | RRSFYVYA | - | - | GMKRSFYVY | HLA-B*15:01 | 6UZS |
|  |  |  |  |  | HLA-A*03:01 | 6O9B |
|  |  |  |  |  | HLA-A*29:02 | - |
|  |  |  |  |  | HLA-A*30:02 | - |
|  |  |  |  |  | HLA-A*31:01 | - |
|  |  |  |  |  | HLA-B*27:05 | \| 5IB2 \| \| --- \| |
|  |  |  |  | SFYVYANGGR | HLA-A*03:01 | 6O9B |
|  |  |  |  |  | HLA-A*11:01 | 4N8V |
|  |  |  |  |  | HLA-A*31:01 | - |
|  |  |  |  |  | HLA-A*33:01 | - |
|  |  |  |  |  | HLA-A*68:01 | 6PBH |
| 9 | SLKELLQN | - | - | AVLKELLQK | HLA-A*11:01 | 4N8V |
|  |  |  |  | AVLKELLQK | HLA-A*11:01 | 4N8V |
|  |  |  |  | QSLKELLQNW | HLA-B*57:01 | 6BXP |
|  |  |  |  | QSLKELLQNW | HLA-B*57:03 | 6V2P |
|  |  |  |  | QSLKELLQNW | HLA-B*58:01 | 5VWH |
|  |  |  |  | IAVLDMCAALKELLQ | HLA-B*57:01 | 6BXP |
|  |  |  |  |  | HLA-B*57:03 | 6V2P |
|  |  |  |  |  | HLA-B*58:01 | 5VWH |
|  |  |  |  | LKELLQNGMNGRTIL | HLA-DRB1*01:01 | - |
| 10 | PGSGVPV | - | - | KEPGSGVPVVL | HLA-B*40:02 | 5IEH |
|  |  |  |  | GSGVPVVL | HLA-C*12:02 | - |
| 11 | RYPANSIV | HLA-A*24:02  HLA-A*24:03  HLA-C*14:02  HLA-C*14:03 | - | YPANSIVVV | HLA-B*51:01 | - |
|  |  |  |  |  | HLA-B*54:01 | - |
|  |  |  |  |  | HLA-B*51:08 | - |
|  |  |  |  | YPANSIVV | HLA-B*51:01 | - |
|  |  |  |  | YPANSIVVV | HLA-B*51:01 | - |
|  |  |  |  | TVTRYPANSIVVVG | HLA-DPA1*02:01 | - |
|  |  |  |  |  | DPB1*10:01 | - |
|  |  |  |  | HSRTVTRYPANSIVV | HLA-DRB5*02:02 | - |
|  |  |  |  | HSRTVTRYPANSIVV | HLA-DRB1*15:01 | - |
|  |  |  |  | HSRTVTRYPANSIVV | HLA-DRB1*15:01 | - |
|  |  |  |  | SRTVTRYPANSIVV | HLA-DRB5*02:02 | - |
| 12 | GPPGTGKS | - |  | LVGPPGTGK | HLA-A*03:01 | 6O9B |
|  |  |  |  | LVGPPGTGK | HLA-A*03:01 | 6O9B |
|  |  |  |  |  | HLA-A*34:02 | - |
|  |  |  |  | YGPPGTGKTL | H2-Kq | - |
|  |  |  |  |  | H2-Dq | - |
|  |  |  |  | YGPPGTGKTL | HLA-C*01:02 | - |
|  |  |  |  | YGPPGTGKTL | HLA-B*35:03 | - |
|  |  |  |  |  | HLA-C*07:04 | - |
|  |  |  |  |  | HLA-C*03:03 | - |
|  |  |  |  |  | HLA-B*18:01 | 6MT3 |
|  |  |  |  |  | HLA-C*01:02 | - |
|  |  |  |  | GPPGTGKTLI | HLA-G*01:01 | - |
|  |  |  |  |  | HLA-A*02:01 | 4U6X |
|  |  |  |  |  | HLA-C*03:03 | - |
|  |  |  |  | GPPGTGKSYLAKAVATEAN | HLA-DRA*01:01 | - |
|  |  |  |  |  | DRB1*08:01 | - |
|  |  |  |  | LTGPPGTGK | HLA-A*34:02 | - |
|  |  |  |  | ILYGPPGTGK | HLA-A*03:01 | 6O9B |
|  |  |  |  |  | HLA-A*34:02 | - |
|  |  |  |  |  | HLA-A*11:01 | 4N8V |
|  |  |  |  |  | HLA-A3 | - |
|  |  |  |  |  | HLA-A*03:01 | 6O9B |
|  |  |  |  | LLYGPPGTGK | HLA-A*03:01 | 6O9B |
|  |  |  |  | LLYGPPGTGK | HLA-A*03:01 | 6O9B |
|  |  |  |  | LLYGPPGTGK | HLA-A*03:01 | 6O9B |
|  |  |  |  | GPPGTGKTAL | HLA-B*27:05 | 6PWY |
|  |  |  |  |  | HLA-B*07:02 | 6AT5 |
|  |  |  |  | LMYGPPGTGK | HLA-A3 | - |
|  |  |  |  | GPPGTGKTI | HLA-C*01:02 | - |
|  |  |  |  |  | HLA-B*42:01 | - |
|  |  |  |  | GPPGTGKTLIARAVANETG | HLA-DQA1*05:05 | - |
|  |  |  |  |  | DQB1*03:01 | - |
|  |  |  |  | GPPGTGKTLIARAVANETG | HLA-DRA*01:01 | - |
|  |  |  |  |  | DRB1*08:01 | - |
|  |  |  |  | IIFGPPGTGK | HLA-A*03:01 | 6O9B |
|  |  |  |  | IIFGPPGTGK | HLA-A*03:01 | 6O9B |
|  |  |  |  | GPPGTGKTL | HLA-C*01:02 | - |
|  |  |  |  | GPPGTGKTLL | HLA-C*01:02 | - |
|  |  |  |  | GPPGTGKTLL | HLA-A*02:01 | 4U6X |
|  |  |  |  | VGPPGTGKTL | HLA-C*01:02 | - |
|  |  |  |  | LLFGPPGTGK | HLA-A*03:01 | 6O9B |
|  |  |  |  | GILLYGPPGTGK | HLA-C*12:02 | - |
|  |  |  |  | GILLYGPPGTGK | HLA-A*11:01 | 4N8V |
|  |  |  |  | GPPGTGKTLIARAVANETGA | HLA-DRA*01:01 | - |
|  |  |  |  |  | DRB1*08:01 | - |
|  |  |  |  | GPPGTGKTLIARAVANETGAF | HLA-DQA1*05:05 | - |
|  |  |  |  |  | DQB1*03:01 | - |
|  |  |  |  | LLYGPPGTGKTLIARAV | HLA-DR | - |
|  |  |  |  | LLYGPPGTGKTLLARAV | HLA-DR | - |
|  |  |  |  | IIHGPPGTGK | HLA-A*03:01 | 6O9B |
|  |  |  |  | LIHGPPGTGK | HLA-A*03:01 | 6O9B |
|  |  |  |  | VPGTGKSTL | HLA-B*07:02 | 6AT5 |
|  |  |  |  | FGPPGTGKTL | HLA-C*01:02 | - |
|  |  |  |  | GVILYGPPGTGK | HLA-C*12:02 | - |
|  |  |  |  | GVLLYGPPGTGK | HLA-C*12:02 | - |
|  |  |  |  | RAVLLAGPPGTGKTALA | HLA-F*01:03 | - |
|  |  |  |  | ILYGPPGTGKTL | HLA-C*03:04 | - |
|  |  |  |  | ILYGPPGTGKTLL | HLA-A*02:02 | - |
|  |  |  |  | LLFGPPGTGKSY | HLA-B*15:01 | 6UZS |
|  |  |  |  | MVGPPGTGK | HLA-A*34:02 | - |
|  |  |  |  | GPPGTGKTVTSATIVYH | HLA-DPA1*02:01 | - |
|  |  |  |  |  | DPB1*14:01 | - |
|  |  |  |  | TPGTGKSKLETLPKEDLIK | HLA-DPA1*02:01 | - |
|  |  |  |  |  | DPB1*13:01 | - |
|  |  |  |  | LLYGPPGTGK | HLA-A*03:01 | 6O9B |
|  |  |  |  |  | HLA-B*07:02 | 6AT5 |
|  |  |  |  |  | HLA-B*35:03 | - |
|  |  |  |  |  | HLA-C*04:01 | - |
|  |  |  |  |  | HLA-C*07:02 | 6AT5 |
|  |  |  |  | VVGPPGTGK | HLA-A*03:01 | 6O9B |
|  |  |  |  | LQGPPGTGK | HLA-A*03:01 | 6O9B |
|  |  |  |  |  | HLA-A*11:01 | 4N8V |
|  |  |  |  |  | HLA-A*31:01 | - |
|  |  |  |  | TLQGPPGTGK | HLA-A*03:01 | 6O9B |
|  |  |  |  |  | HLA-A*11:01 | 4N8V |
|  |  |  |  |  | HLA-A*31:01 | - |
|  |  |  |  |  | HLA-A*68:01 | 6PBH |
| 13 | NVAITRAK | HLA-A*34:02 | - | RRLNVAITR | HLA-B*27:05 | - |
|  |  |  |  | RFNVAITRAK | - | - |
|  |  |  |  | VNRFNVAITRAKIGI | - | - |
|  |  |  |  | NANRFNVAITRAKKG | - | - |
| 14 | QGPPGTGK | - | - | RAVLLAGPPGTGKTALA | HLA-F*01:03 | - |
|  |  |  |  | LLYGPPGTGK | HLA-A*03:01 | 6O9B |
|  |  |  |  | LLYGPPGTGK | HLA-A*03:01 | 6O9B |
|  |  |  |  | LLYGPPGTGK | HLA-A*03:01 | 6O9B |
|  |  |  |  | ILYGPPGTGK | HLA-A*03:01 | 6O9B |
|  |  |  |  |  | HLA-A*11:01 | 4N8V |
|  |  |  |  | GPPGTGKTAL | HLA-B*27:05 | 5IB2 |
|  |  |  |  |  | HLA-B*07:02 | 6AT5 |
|  |  |  |  | GPPGTGKTAL | HLA-B*27:05 | 5IB2 |
|  |  |  |  | LVGPPGTGK | HLA-A*03:01 | 6O9B |
|  |  |  |  |  | HLA-A*34:02 | - |
|  |  |  |  | LVGPPGTGK | HLA-A*03:01 | 6O9B |
|  |  |  |  | YGPPGTGKTL | H2-Kq | - |
|  |  |  |  |  | H2-Dq | - |
|  |  |  |  | YGPPGTGKTL | HLA-C*01:02 | - |
|  |  |  |  | YGPPGTGKTL | HLA-B*35:03 | - |
|  |  |  |  |  | HLA-C*07:04 | - |
|  |  |  |  |  | HLA-C*03:03 | - |
|  |  |  |  |  | HLA-B*18:01 | 6MT3 |
|  |  |  |  |  | HLA-C*01:02 | - |
|  |  |  |  | LMYGPPGTGK | HLA-A3 | - |
|  |  |  |  | MVGPPGTGK | HLA-A*68:01 | 6MT3 |
|  |  |  |  |  | HLA-A*34:01 | - |
|  |  |  |  |  | HLA-A*34:02 | - |
|  |  |  |  | GPPGTGKTI | HLA-C*01:02 | - |
|  |  |  |  |  | HLA-B*42:0 | - |
|  |  |  |  | GPPGTGKTLIARAVANETG | HLA-DQA1*05:05 | - |
|  |  |  |  |  | DQB1*03:01 | - |
|  |  |  |  |  | HLA-DRA*01:01 | - |
|  |  |  |  |  | DQB1*08:01 | - |
|  |  |  |  | IIFGPPGTGK | HLA-A*03:01 | 6O9B |
|  |  |  |  | IIFGPPGTGK | HLA-A*03:01 | 6O9B |
|  |  |  |  | VVGPPGTGK | HLA-A*03:01 | 6O9B |
|  |  |  |  | GPPGTGKTLI | HLA-G*01:01 | - |
|  |  |  |  |  | HLA-A*02:01 | 4U6X |
|  |  |  |  |  | HLA-C*03:03 | - |
|  |  |  |  | GPPGTGKSYLAKAVATEAN | HLA-DRA*01:01 | - |
|  |  |  |  |  | DRB1*08:01 | - |
|  |  |  |  | LTGPPGTGK | HLA-A*34:02 | - |
|  |  |  |  | GPPGTGKTL | HLA-C*01:02 | - |
|  |  |  |  | GPPGTGKTLL | HLA-C*01:02 | - |
|  |  |  |  | GPPGTGKTLL | HLA-A*02:01 | 4U6X |
|  |  |  |  | VGPPGTGKTL | HLA-C*01:02 | - |
|  |  |  |  | GILLYGPPGTGK | HLA-C*12:02 | - |
|  |  |  |  | GILLYGPPGTGK | HLA-A*11:01 | 4N8V |
|  |  |  |  | GPPGTGKTLIARAVANETGA | HLA-DRA*01:01 | - |
|  |  |  |  |  | DRB1*08:01 | - |
|  |  |  |  | GPPGTGKTLIARAVANETGAF | HLA-DQA1*05:05 | - |
|  |  |  |  |  | DQB1*03:01 | - |
|  |  |  |  | LLYGPPGTGKTLIARAV | HLA-DR | - |
|  |  |  |  | LLYGPPGTGKTLLARAV | HLA-DR | - |
|  |  |  |  | FGPPGTGKTL | HLA-C*01:02 | - |
|  |  |  |  | GVILYGPPGTGK | HLA-C*12:02 | - |
|  |  |  |  | GVLLYGPPGTGK | HLA-C*12:02 | - |
|  |  |  |  | ILYGPPGTGKTL | HLA-C*03:04 | - |
|  |  |  |  | ILYGPPGTGKTLL | HLA-A*02:02 | - |
|  |  |  |  | LLFGPPGTGKSY | HLA-B*15:01 | 6UZS |
|  |  |  |  | GPPGTGKTVTSATIVYH | HLA-DPA1*02:01 | - |
|  |  |  |  |  | DPB1*14:01 | - |
|  |  |  |  | LLFGPPGTGK | HLA-A*03:01 | 6O9B |
|  |  |  |  | LQGPPGTGK | HLA-A*03:01 | 6O9B |
|  |  |  |  |  | HLA-A*11:01 | 4N8V |
|  |  |  |  |  | HLA-A*31:01 | - |
|  |  |  |  | TLQGPPGTGK | HLA-A*03:01 | 6O9B |
|  |  |  |  |  | HLA-A*11:01 | 4N8V |
|  |  |  |  |  | HLA-A*31:01 | - |
|  |  |  |  |  | HLA-A*68:01 | 6PBH |
| 15 | RFNVAITR | - | - | RFNVAITRAK | HLA-A*03:01 | 6O9B |
|  |  |  |  |  | HLA-A*11:01 | 4N8V |
|  |  |  |  |  | HLA-A*31:01 | - |
|  |  |  |  |  | HLA-A*68:01 | 6PBH |
|  |  |  |  |  | HLA-DRB1*01:01 | - |
|  |  |  |  | NANRFNVAITRAKKG | - | - |
|  |  |  |  | NVNRFNVAITRAKVG | - | - |
| 16 | VTLIGEAV | - | - | SLSALIGEAV | HLA-A*02:01 | 4U6X |
|  |  |  |  | VPVTLIGEAVF | HLA-B*35:01 | 4PRN |
|  |  |  |  |  | HLA-H2-Dq | - |
|  |  |  |  | VPVTLIGEA | HLA-B*55:01 | - |
|  |  |  |  |  | HLA-H2-Dq | - |
|  |  |  |  | GQKGTGKWTAISALE | HLA-B*15:01 | 6UZS |
|  |  |  |  | ALIGEAVGARLI | HLA-B*13:01 | - |
|  |  |  |  | APSLSALIGEAVGAR | HLA-DR1 | - |
| 17 | LALITLAT | - | - | LITLATCELYHYQECV | - | - |
|  |  |  |  | LITLATCELYHYQECVR | - | - |
|  |  |  |  | MKIILFLALITLATC | - | - |
| 18 | DEDDSEPV | - | - | VVFDDSEPVQK | HLA-A*11:01 | 4N8V |
|  |  |  |  | VVFDDSEPVQRIL | Mamu B*008:01 | - |
|  |  |  |  |  | HLA-B*27:05 | 5IB2 |
|  |  |  |  | VVFDDSEPV | HLA-C*08:01 | - |
|  |  |  |  | DDSEPVLKGVKLHYT | HLA-DRB1*01:01 | - |
|  |  |  |  | CKFDEDDSEPVLKGVKLHYT | - | - |
|  |  |  |  | DEDDSEPVL | HLA-B*40:01 | - |
|  |  |  |  |  | HLA-B*40:02 | 5IEH |
|  |  |  |  | FDEDDSEPVLKGVKLHYT | - | - |
|  |  |  |  | GCCSCGSCCKFDEDDSEPVL | - | - |
|  |  |  |  | CCSCGSCCKFDEDDSEPVLKGVKL | HLA-B*40:01 | - |
|  |  |  |  | FDEDDSEPVL | - | - |
| 19 | RRARSVAS | - | - | DHSPRARSVAP | HLA-A*66:01 | - |
|  |  |  |  | SPRRARSV | HLA-B*08:01 | 3X13 |
|  |  |  |  | AGCLIGAEHVNNSYECDIPIGAGICASYQTQTNSPRRARSVAS | HLA class I | - |
|  |  |  |  | ARSVASQSIIAYTMSLGAENSVAYSNNSIAIPTNFTISVTTEI | HLA class I | - |
|  |  |  |  | SYQTQTNSPRRARSVA | HLA class I | - |
|  |  |  |  | QTQTNSPRRARSVAS | - | - |
| 20 | VFLLVTLA | - | - | LRVDPVNFKLLSHCLLVTLA | HLA-DPA1*01:03 | - |
|  |  |  |  |  | DPB1*04:02 | - |
|  |  |  |  | LLSHSLLVTLA | HLA-A*02:02 | - |
|  |  |  |  |  | HLA-A*02:05 | - |
|  |  |  |  | DPVNFKLLSHCLLVTLAAH | HLA-DPA1*01:03 | - |
|  |  |  |  |  | DPB1*04:02 | - |
|  |  |  |  | FLLVTLAIL | HLA-A*02:01 | 4U6X |
|  |  |  |  |  | HLA-A*02:02 | - |
|  |  |  |  |  | HLA-A*02:03 | 3OX8 |
|  |  |  |  |  | HLA-A*02:06 | 3OXR |
|  |  |  |  |  | HLA-A*68:02 | - |
|  |  |  |  | FLLVTLAILTALRLC | HLA-DRB1*01:01 | - |
|  |  |  |  | FVVFLLVTL | HLA-A*02:01 | 4U6X |
|  |  |  |  |  | HLA-A*02:02 | - |
|  |  |  |  |  | HLA-A*02:03 | 3OX8 |
|  |  |  |  |  | HLA-A*02:06 | 3OXR |
|  |  |  |  |  | HLA-A*68:02 | - |
|  |  |  |  | LLVTLAILTA | HLA-A*02:01 | 4U6X |
|  |  |  |  |  | HLA-A*02:02 | - |
|  |  |  |  |  | HLA-A*02:03 | 3OX8 |
|  |  |  |  |  | HLA-A*02:06 | 3OXR |
|  |  |  |  |  | HLA-A*68:02 | - |
|  |  |  |  | AFVVFLLVTLAILTA | HLA-DRB1*01:01 | - |
|  |  |  |  | FLAFVVFLLVTLAIL | HLA-DRB1*01:01 |  |
|  |  |  |  | LAFVVFLLVTLAILT | HLA-DRB1*01:01 | - |
|  |  |  |  | SVLLFLAFVVFLLVT | HLA-DRB1*01:01 | - |
|  |  |  |  | VFLLVTLAILTALRL | HLA-DRB1*01:01 | - |
|  |  |  |  | VLLFLAFVVFLLVTL | HLA-DRB1*01:01 | - |
|  |  |  |  | VVFLLVTLAILTALR | HLA-DRB1*01:01 | - |
| 21 | VNSVLLFL | - | - | RSVNSVLLF | HLA-C*15:05 | - |
|  |  |  |  |  | HLA-B*58:01 | 5VWH |
|  |  |  |  |  | HLA-B*57:03 | 6V2P |
|  |  |  |  |  | HLA-B*15:01 | - |
|  |  |  |  |  | HLA-B*57:01 | 6BXP |
|  |  |  |  | MELSVLLFL | HLA-A*02:01 | 4U6X |
|  |  |  |  | TVNSVLLFL | HLA-C*15:02 | - |
|  |  |  |  | RSVNSVLLF | HLA-B*58:01 | 5VWH |
|  |  |  |  | LIVNSVLLFL | HLA-A*02:01 | 4U6X |
|  |  |  |  |  | HLA-A*02:02 | - |
|  |  |  |  |  | HLA-A*02:03 | 3Ox8 |
|  |  |  |  |  | HLA-A*02:06 | 3OXR |
|  |  |  |  |  | HLA-A*68:02 | - |
|  |  |  |  | SVLLFLAFV | HLA-A*02:01 | 4U6X |
|  |  |  |  |  | HLA-A*02:02 | - |
|  |  |  |  |  | HLA-A*02:03 | 3OX8 |
|  |  |  |  |  | HLA-A*02:06 | 3OXR |
|  |  |  |  |  | HLA-A*68:02 | - |
|  |  |  |  | IVNSVLLFL | HLA-A*02:01 | 4U6X |
|  |  |  |  |  | HLA-A*02:02 | - |
|  |  |  |  |  | HLA-A*02:03 | 3OX8 |
|  |  |  |  |  | HLA-A*02:06 | 3OXR |
|  |  |  |  |  | HLA-A*68:02 | - |
|  |  |  |  | SVLLFLAFVV | HLA-A*02:01 | 4U6X |
|  |  |  |  |  | HLA-A*02:02 | - |
|  |  |  |  |  | HLA-A*02:03 | 3OX8 |
|  |  |  |  |  | HLA-A*02:06 | 3OXR |
|  |  |  |  |  | HLA-A*68:02 | - |
|  |  |  |  | GTLIVNSVLLFLAFV | HLA-DRB1*01:01 | - |
|  |  |  |  | SVLLFLAFVVFLLVT | HLA-DRB1*01:01 | - |
|  |  |  |  | TGTLIVNSVLLFLAF | HLA-DRB1*01:01 | - |
|  |  |  |  | TLIVNSVLLFLAFVV | HLA-DRB1*01:01 | - |
|  |  |  |  | VNSVLLFLAFVVFLL | HLA-DRB1*01:01 | - |
|  |  |  |  | SEETGTLIVNSVLLF | - | - |
