# Supplementary material for: SARS-CoV-2 Proteome Harbors Peptides Which Are Able to Trigger Autoimmunity Responses: Implications for Infection, Vaccination, and Population Coverage
Source: Front Immunol. 2021 Aug 10;12:705772. doi: 10.3389/fimmu.2021.705772 (PMC8383889; doi:10.3389/fimmu.2021.705772)
Supplement: Supplementary file 6 [file Table_4.docx]

Supplementary Table 4. The population coverage calculation for SARS-CoV-2 peptides and their equivalent IEDB peptides. (a: projected population coverage, b: average number of epitope hits / HLA combinations recognized by the population, and c: minimum number of epitope hits / HLA combinations recognize).

| **population/area** | **Coverage ^a^** | **population/area** | **Coverage ^a^** |
| --- | --- | --- | --- |
| Average | 50.14 | [Croatia](http://tools.iedb.org/population/result/#Croatia) | 51.29% |
| Standard deviation | 23.46 | [Croatia Caucasoid](http://tools.iedb.org/population/result/#Croatia Caucasoid) | 51.29% |
| [Papua New Guinea](http://tools.iedb.org/population/result/#Papua New Guinea) | 97.59% | [Chile](http://tools.iedb.org/population/result/#Chile) | 51.10% |
| [Papua New Guinea Melanesian](http://tools.iedb.org/population/result/#Papua New Guinea Melanesian) | 97.59% | [Germany](http://tools.iedb.org/population/result/#Germany) | 50.90% |
| [New Caledonia](http://tools.iedb.org/population/result/#New Caledonia) | 94.73% | [Germany Caucasoid](http://tools.iedb.org/population/result/#Germany Caucasoid) | 50.90% |
| [New Caledonia Melanesian](http://tools.iedb.org/population/result/#New Caledonia Melanesian) | 94.73% | [Mexico Amerindian](http://tools.iedb.org/population/result/#Mexico Amerindian) | 50.84% |
| [Malaysia Austronesian](http://tools.iedb.org/population/result/#Malaysia Austronesian) | 94.24% | [Brazil Caucasoid](http://tools.iedb.org/population/result/#Brazil Caucasoid) | 50.58% |
| [Chile Amerindian](http://tools.iedb.org/population/result/#Chile Amerindian) | 93.15% | [France](http://tools.iedb.org/population/result/#France) | 50.35% |
| [Indonesia](http://tools.iedb.org/population/result/#Indonesia) | 89.80% | [France Caucasoid](http://tools.iedb.org/population/result/#France Caucasoid) | 50.35% |
| [Indonesia Austronesian](http://tools.iedb.org/population/result/#Indonesia Austronesian) | 89.80% | [Mexico Mestizo](http://tools.iedb.org/population/result/#Mexico Mestizo) | 49.97% |
| [Malaysia](http://tools.iedb.org/population/result/#Malaysia) | 88.21% | [Saudi Arabia](http://tools.iedb.org/population/result/#Saudi Arabia) | 49.66% |
| [Malaysia Oriental](http://tools.iedb.org/population/result/#Malaysia Oriental) | 87.10% | [Saudi Arabia Arab](http://tools.iedb.org/population/result/#Saudi Arabia Arab) | 49.66% |
| [Taiwan](http://tools.iedb.org/population/result/#Taiwan) | 86.40% | [Israel Arab](http://tools.iedb.org/population/result/#Israel Arab) | 48.81% |
| [Taiwan Oriental](http://tools.iedb.org/population/result/#Taiwan Oriental) | 86.40% | [Bulgaria](http://tools.iedb.org/population/result/#Bulgaria) | 48.71% |
| [Japan](http://tools.iedb.org/population/result/#Japan) | 84.88% | [South Africa](http://tools.iedb.org/population/result/#South Africa) | 48.34% |
| [Japan Oriental](http://tools.iedb.org/population/result/#Japan Oriental) | 84.88% | [Portugal](http://tools.iedb.org/population/result/#Portugal) | 47.29% |
| [Singapore Austronesian](http://tools.iedb.org/population/result/#Singapore Austronesian) | 84.36% | [Portugal Caucasoid](http://tools.iedb.org/population/result/#Portugal Caucasoid) | 47.29% |
| [Korea; South](http://tools.iedb.org/population/result/#Korea; South) | 84.28% | [Bulgaria Caucasoid](http://tools.iedb.org/population/result/#Bulgaria Caucasoid) | 47.27% |
| [Korea; South Oriental](http://tools.iedb.org/population/result/#Korea; South Oriental) | 84.28% | [Morocco Arab](http://tools.iedb.org/population/result/#Morocco Arab) | 47.17% |
| [Southeast Asia](http://tools.iedb.org/population/result/#Southeast Asia) | 84.12% | [Austria](http://tools.iedb.org/population/result/#Austria) | 47.03% |
| [East Asia](http://tools.iedb.org/population/result/#East Asia) | 83.78% | [Austria Caucasoid](http://tools.iedb.org/population/result/#Austria Caucasoid) | 47.03% |
| [United States Polynesian](http://tools.iedb.org/population/result/#United States Polynesian) | 82.17% | [Mexico](http://tools.iedb.org/population/result/#Mexico) | 45.89% |
| [Vietnam](http://tools.iedb.org/population/result/#Vietnam) | 82.11% | [Israel](http://tools.iedb.org/population/result/#Israel) | 45.74% |
| [Vietnam Oriental](http://tools.iedb.org/population/result/#Vietnam Oriental) | 82.11% | [Cape Verde](http://tools.iedb.org/population/result/#Cape Verde) | 45.71% |
| [Hong Kong](http://tools.iedb.org/population/result/#Hong Kong) | 81.97% | [Cape Verde Black](http://tools.iedb.org/population/result/#Cape Verde Black) | 45.71% |
| [Hong Kong Oriental](http://tools.iedb.org/population/result/#Hong Kong Oriental) | 81.97% | [Cuba Caucasoid](http://tools.iedb.org/population/result/#Cuba Caucasoid) | 45.46% |
| [Singapore](http://tools.iedb.org/population/result/#Singapore) | 81.73% | [Venezuela Amerindian](http://tools.iedb.org/population/result/#Venezuela Amerindian) | 45.41% |
| [United States Asian](http://tools.iedb.org/population/result/#United States Asian) | 81.54% | [West Africa](http://tools.iedb.org/population/result/#West Africa) | 44.97% |
| [Oceania](http://tools.iedb.org/population/result/#Oceania) | 80.72% | [Uganda](http://tools.iedb.org/population/result/#Uganda) | 44.57% |
| [Singapore Oriental](http://tools.iedb.org/population/result/#Singapore Oriental) | 80.72% | [Uganda Black](http://tools.iedb.org/population/result/#Uganda Black) | 44.57% |
| [South Africa Other](http://tools.iedb.org/population/result/#South Africa Other) | 79.84% | [Venezuela](http://tools.iedb.org/population/result/#Venezuela) | 44.03% |
| [Northeast Asia](http://tools.iedb.org/population/result/#Northeast Asia) | 75.83% | [Southwest Asia](http://tools.iedb.org/population/result/#Southwest Asia) | 43.90% |
| [United States Amerindian](http://tools.iedb.org/population/result/#United States Amerindian) | 75.69% | [West Indies](http://tools.iedb.org/population/result/#West Indies) | 43.78% |
| [American Samoa](http://tools.iedb.org/population/result/#American Samoa) | 75.00% | [Cuba](http://tools.iedb.org/population/result/#Cuba) | 43.06% |
| [American Samoa Polynesian](http://tools.iedb.org/population/result/#American Samoa Polynesian) | 75.00% | [Israel Jew](http://tools.iedb.org/population/result/#Israel Jew) | 42.79% |
| [China](http://tools.iedb.org/population/result/#China) | 74.49% | [Italy](http://tools.iedb.org/population/result/#Italy) | 41.63% |
| [China Oriental](http://tools.iedb.org/population/result/#China Oriental) | 74.49% | [Italy Caucasoid](http://tools.iedb.org/population/result/#Italy Caucasoid) | 41.63% |
| [Russia Siberian](http://tools.iedb.org/population/result/#Russia Siberian) | 73.95% | [United States Black](http://tools.iedb.org/population/result/#United States Black) | 41.09% |
| [Philippines](http://tools.iedb.org/population/result/#Philippines) | 73.50% | [Sao Tome and Principe](http://tools.iedb.org/population/result/#Sao Tome and Principe) | 40.64% |
| [Philippines Austronesian](http://tools.iedb.org/population/result/#Philippines Austronesian) | 73.50% | [Sao Tome and Principe Black](http://tools.iedb.org/population/result/#Sao Tome and Principe Black) | 40.64% |
| [Thailand](http://tools.iedb.org/population/result/#Thailand) | 71.19% | [Morocco](http://tools.iedb.org/population/result/#Morocco) | 40.49% |
| [Thailand Oriental](http://tools.iedb.org/population/result/#Thailand Oriental) | 71.19% | [Cuba Mulatto](http://tools.iedb.org/population/result/#Cuba Mulatto) | 38.79% |
| [Russia](http://tools.iedb.org/population/result/#Russia) | 67.70% | [North Africa](http://tools.iedb.org/population/result/#North Africa) | 37.84% |
| [Australia Australian Aborigines](http://tools.iedb.org/population/result/#Australia Australian Aborigines) | 65.83% | [Tunisia](http://tools.iedb.org/population/result/#Tunisia) | 37.45% |
| [Sweden](http://tools.iedb.org/population/result/#Sweden) | 65.77% | [Tunisia Arab](http://tools.iedb.org/population/result/#Tunisia Arab) | 37.45% |
| [Sweden Caucasoid](http://tools.iedb.org/population/result/#Sweden Caucasoid) | 65.77% | [Senegal](http://tools.iedb.org/population/result/#Senegal) | 37.38% |
| [Ecuador](http://tools.iedb.org/population/result/#Ecuador) | 64.84% | [Senegal Black](http://tools.iedb.org/population/result/#Senegal Black) | 37.38% |
| [Ecuador Amerindian](http://tools.iedb.org/population/result/#Ecuador Amerindian) | 64.84% | [Central Africa](http://tools.iedb.org/population/result/#Central Africa) | 36.99% |
| [South Asia](http://tools.iedb.org/population/result/#South Asia) | 64.04% | [Sudan Mixed](http://tools.iedb.org/population/result/#Sudan Mixed) | 36.58% |
| [Pakistan Mixed](http://tools.iedb.org/population/result/#Pakistan Mixed) | 63.53% | [Cameroon](http://tools.iedb.org/population/result/#Cameroon) | 36.45% |
| [India](http://tools.iedb.org/population/result/#India) | 62.42% | [Cameroon Black](http://tools.iedb.org/population/result/#Cameroon Black) | 36.45% |
| [India Asian](http://tools.iedb.org/population/result/#India Asian) | 62.42% | [Sudan](http://tools.iedb.org/population/result/#Sudan) | 36.07% |
| [Bulgaria Other](http://tools.iedb.org/population/result/#Bulgaria Other) | 62.39% | [Jordan](http://tools.iedb.org/population/result/#Jordan) | 35.88% |
| [Australia](http://tools.iedb.org/population/result/#Australia) | 62.37% | [Jordan Arab](http://tools.iedb.org/population/result/#Jordan Arab) | 35.88% |
| [Argentina](http://tools.iedb.org/population/result/#Argentina) | 60.61% | [Morocco Caucasoid](http://tools.iedb.org/population/result/#Morocco Caucasoid) | 35.18% |
| [Argentina Amerindian](http://tools.iedb.org/population/result/#Argentina Amerindian) | 60.61% | [Mali](http://tools.iedb.org/population/result/#Mali) | 34.52% |
| [Iran](http://tools.iedb.org/population/result/#Iran) | 60.40% | [Mali Black](http://tools.iedb.org/population/result/#Mali Black) | 34.52% |
| [Iran Persian](http://tools.iedb.org/population/result/#Iran Persian) | 60.40% | [East Africa](http://tools.iedb.org/population/result/#East Africa) | 31.42% |
| [Pakistan](http://tools.iedb.org/population/result/#Pakistan) | 60.15% | [Czech Republic](http://tools.iedb.org/population/result/#Czech Republic) | 30.69% |
| [Australia Caucasoid](http://tools.iedb.org/population/result/#Australia Caucasoid) | 60.01% | [Czech Republic Caucasoid](http://tools.iedb.org/population/result/#Czech Republic Caucasoid) | 30.69% |
| [Brazil Amerindian](http://tools.iedb.org/population/result/#Brazil Amerindian) | 58.78% | [Chile Mixed](http://tools.iedb.org/population/result/#Chile Mixed) | 29.96% |
| [Pakistan Asian](http://tools.iedb.org/population/result/#Pakistan Asian) | 58.51% | [Kenya](http://tools.iedb.org/population/result/#Kenya) | 29.33% |
| [Georgia Caucasoid](http://tools.iedb.org/population/result/#Georgia Caucasoid) | 58.34% | [Kenya Black](http://tools.iedb.org/population/result/#Kenya Black) | 29.33% |
| [Ireland South](http://tools.iedb.org/population/result/#Ireland South) | 58.28% | [South Africa Black](http://tools.iedb.org/population/result/#South Africa Black) | 26.79% |
| [Ireland South Caucasoid](http://tools.iedb.org/population/result/#Ireland South Caucasoid) | 58.28% | [Spain](http://tools.iedb.org/population/result/#Spain) | 26.58% |
| [Peru](http://tools.iedb.org/population/result/#Peru) | 57.57% | [Spain Caucasoid](http://tools.iedb.org/population/result/#Spain Caucasoid) | 26.58% |
| [Peru Amerindian](http://tools.iedb.org/population/result/#Peru Amerindian) | 57.57% | [Zimbabwe](http://tools.iedb.org/population/result/#Zimbabwe) | 23.15% |
| [Mongolia](http://tools.iedb.org/population/result/#Mongolia) | 57.43% | [Zimbabwe Black](http://tools.iedb.org/population/result/#Zimbabwe Black) | 23.15% |
| [Mongolia Oriental](http://tools.iedb.org/population/result/#Mongolia Oriental) | 57.43% | [Serbia](http://tools.iedb.org/population/result/#Serbia) | 22.03% |
| [World](http://tools.iedb.org/population/result/#World) | 57.41% | [Serbia Caucasoid](http://tools.iedb.org/population/result/#Serbia Caucasoid) | 22.03% |
| [United States](http://tools.iedb.org/population/result/#United States) | 57.12% | [Scotland](http://tools.iedb.org/population/result/#Scotland) | 20.93% |
| [North America](http://tools.iedb.org/population/result/#North America) | 57.02% | [Scotland Caucasoid](http://tools.iedb.org/population/result/#Scotland Caucasoid) | 20.93% |
| [Romania](http://tools.iedb.org/population/result/#Romania) | 57.00% | [Sri Lanka](http://tools.iedb.org/population/result/#Sri Lanka) | 19.72% |
| [Romania Caucasoid](http://tools.iedb.org/population/result/#Romania Caucasoid) | 57.00% | [Sri Lanka Asian](http://tools.iedb.org/population/result/#Sri Lanka Asian) | 19.72% |
| [England](http://tools.iedb.org/population/result/#England) | 56.69% | [Zambia](http://tools.iedb.org/population/result/#Zambia) | 19.25% |
| [England Caucasoid](http://tools.iedb.org/population/result/#England Caucasoid) | 56.69% | [Zambia Black](http://tools.iedb.org/population/result/#Zambia Black) | 19.25% |
| [Georgia](http://tools.iedb.org/population/result/#Georgia) | 56.51% | [Macedonia](http://tools.iedb.org/population/result/#Macedonia) | 18.55% |
| [Finland](http://tools.iedb.org/population/result/#Finland) | 56.02% | [Macedonia Caucasoid](http://tools.iedb.org/population/result/#Macedonia Caucasoid) | 18.55% |
| [Finland Caucasoid](http://tools.iedb.org/population/result/#Finland Caucasoid) | 56.02% | [Sudan Arab](http://tools.iedb.org/population/result/#Sudan Arab) | 18.50% |
| [Belgium](http://tools.iedb.org/population/result/#Belgium) | 55.60% | [Central African Republic](http://tools.iedb.org/population/result/#Central African Republic) | 7.99% |
| [Belgium Caucasoid](http://tools.iedb.org/population/result/#Belgium Caucasoid) | 55.60% | [Central African Republic Black](http://tools.iedb.org/population/result/#Central African Republic Black) | 7.99% |
| [Brazil Mixed](http://tools.iedb.org/population/result/#Brazil Mixed) | 55.42% | [Switzerland](http://tools.iedb.org/population/result/#Switzerland) | 7.46% |
| [Brazil](http://tools.iedb.org/population/result/#Brazil) | 55.32% | [Switzerland Caucasoid](http://tools.iedb.org/population/result/#Switzerland Caucasoid) | 7.46% |
| [South America](http://tools.iedb.org/population/result/#South America) | 55.29% | [Ivory Coast](http://tools.iedb.org/population/result/#Ivory Coast) | 6.67% |
| [Ireland Northern](http://tools.iedb.org/population/result/#Ireland Northern) | 55.21% | [Ivory Coast Black](http://tools.iedb.org/population/result/#Ivory Coast Black) | 6.67% |
| [Ireland Northern Caucasoid](http://tools.iedb.org/population/result/#Ireland Northern Caucasoid) | 55.21% | [Burkina Faso](http://tools.iedb.org/population/result/#Burkina Faso) | 6.57% |
| [Russia Other](http://tools.iedb.org/population/result/#Russia Other) | 55.20% | [Burkina Faso Black](http://tools.iedb.org/population/result/#Burkina Faso Black) | 6.57% |
| [Poland](http://tools.iedb.org/population/result/#Poland) | 54.32% | [Martinique](http://tools.iedb.org/population/result/#Martinique) | 5.91% |
| [Poland Caucasoid](http://tools.iedb.org/population/result/#Poland Caucasoid) | 54.32% | [Martinique Black](http://tools.iedb.org/population/result/#Martinique Black) | 5.91% |
| [Georgia Kurd](http://tools.iedb.org/population/result/#Georgia Kurd) | 54.11% | [Rwanda](http://tools.iedb.org/population/result/#Rwanda) | 2.97% |
| [Guinea-Bissau](http://tools.iedb.org/population/result/#Guinea-Bissau) | 53.75% | [Rwanda Black](http://tools.iedb.org/population/result/#Rwanda Black) | 2.97% |
| [Guinea-Bissau Black](http://tools.iedb.org/population/result/#Guinea-Bissau Black) | 53.75% | [Venezuela Caucasoid](http://tools.iedb.org/population/result/#Venezuela Caucasoid) | 2.39% |
| [United States Caucasoid](http://tools.iedb.org/population/result/#United States Caucasoid) | 53.35% | [United Kingdom](http://tools.iedb.org/population/result/#United Kingdom) | 2.19% |
| [United States Mestizo](http://tools.iedb.org/population/result/#United States Mestizo) | 52.82% | [United Kingdom Caucasoid](http://tools.iedb.org/population/result/#United Kingdom Caucasoid) | 2.19% |
| [Oman](http://tools.iedb.org/population/result/#Oman) | 52.61% | [Lebanon](http://tools.iedb.org/population/result/#Lebanon) | 1.99% |
| [Oman Arab](http://tools.iedb.org/population/result/#Oman Arab) | 52.61% | [Lebanon Mixed](http://tools.iedb.org/population/result/#Lebanon Mixed) | 1.99% |
| [Europe](http://tools.iedb.org/population/result/#Europe) | 52.24% | [Venezuela Mestizo](http://tools.iedb.org/population/result/#Venezuela Mestizo) | 0.00% |
| [United States Hispanic](http://tools.iedb.org/population/result/#United States Hispanic) | 51.77% |  |  |
